# Large Enrichment of Anthropogenic Organic Matter Degrading Bacteria in the Sea-Surface Microlayer at Coastal Livingston Island (Antarctica)

**DOI:** 10.3389/fmicb.2020.571983

**Published:** 2020-09-11

**Authors:** Alícia Martinez-Varela, Gemma Casas, Benjamin Piña, Jordi Dachs, Maria Vila-Costa

**Affiliations:** Department of Environmental Chemistry, Institut de Diagnóstic Ambiental i Estudis de l’aigua, Consejo Superior de Investigaciones Científicas (IDAEA-CSIC), Barcelona, Spain

**Keywords:** sea-surface microlayer, microbial communities, organic pollutants, surfactants, amplicon sequencing, bacterioneuston, ADOC

## Abstract

The composition of bacteria inhabiting the sea-surface microlayer (SML) is poorly characterized globally and yet undescribed for the Southern Ocean, despite their relevance for the biogeochemistry of the surface ocean. We report the abundances and diversity of bacteria inhabiting the SML and the subsurface waters (SSL) determined from a unique sample set from a polar coastal ecosystem (Livingston Island, Antarctica). From early to late austral summer (January–March 2018), we consistently found a higher abundance of bacteria in the SML than in the SSL. The SML was enriched in some Gammaproteobacteria genus such as *Pseudoalteromonas*, *Pseudomonas*, and *Colwellia*, known to degrade a wide range of semivolatile, hydrophobic, and surfactant-like organic pollutants. Hydrocarbons and other synthetic chemicals including surfactants, such as perfluoroalkyl substances (PFAS), reach remote marine environments by atmospheric transport and deposition and by oceanic currents, and are known to accumulate in the SML. Relative abundances of specific SML-enriched bacterial groups were significantly correlated to concentrations of PFASs, taken as a proxy of hydrophobic anthropogenic pollutants present in the SML and its stability. Our observations provide evidence for an important pollutant-bacteria interaction in the marine SML. Given that pollutant emissions have increased during the Anthropocene, our results point to the need to assess chemical pollution as a factor modulating marine microbiomes in the contemporaneous and future oceans.

## Introduction

The sea surface microlayer (SML) is the uppermost top layer of the marine water column and constitutes the boundary layer where gases and particles exchange between the sea and the atmosphere (Garbe et al., 2014). As a microenvironment thinner than 1000 μm, the SML exhibits distinct, extreme and variable physicochemical and biological features compared to those in the underlying bulk water. The structure and function of SML bacterial residents, known as bacterioneuston (Naumann, 1917), have been poorly characterized, probably due to logistical limitations and the difficulties for sampling the SML. However, the bacterioneuston is thought to play a central role in the mediation of many air-sea exchange interactions and biogeochemical and climate-related processes at regional and global scales (Kuznetsova and Lee, 2001; Brooks et al., 2009; Nakajima et al., 2013). Far from being a benevolent habitat for microbial life, bacterioneuston withstand intense environmental stressors such as harsh ultra-violet (UV) radiation, wind speed driven turbulences, temperature and salinity gradients, as well as the enrichment of toxic persistent compounds such as heavy metals or hydrophobic organic pollutants (OP) (Guerin, 1989; Wurl and Obbard, 2004; Agogué et al., 2005; Cincinelli et al., 2005; García-Flor et al., 2005; Ju et al., 2008; Wurl and Holmes, 2008; Stolle et al., 2011; Cunliffe et al., 2013; Engel et al., 2018; Rahlff et al., 2019). In the SML, in addition to an accumulation of hydrophobic organic compounds, there is an enrichment of organic matter (OM) with surfactant properties, both anthropogenic and biogenic. The accumulation of these pools of OM in the SML, which under calm meteorological conditions can even form visible aggregates or surface slicks, can promote bacterial growth (Stolle et al., 2011; Cunliffe et al., 2013; Kurata et al., 2016; Sabbaghzadeh et al., 2017; Engel et al., 2018; Parks et al., 2020). For instance, fast peptide turnovers (Kuznetsova and Lee, 2001) or degradation of hydrocarbons (Coelho et al., 2011) have been observed in the SML.

In terms of community composition, high similarity between subsurface layer (SSL) and SML bacterial community structure have been observed in a number of oceanic regions (Agogué et al., 2005; Obernosterer et al., 2005; Fan et al., 2018; Zäncker et al., 2018), possibly caused by disruption of the SML and mixing with underlying waters. Uncoupling between bacterioplankton and bacterioneuston community compositions have been observed under low wind speeds conditions and visible surface slicks (Stolle et al., 2010). As bacterioneuston communities can be transferred from the SML into the atmosphere through sea-spray aerosol, then subject to atmospheric transport, the composition of the SML is a key factor for the regional and global microbial dispersion (Aller et al., 2005; Hervas and Casamayor, 2009; Mayol et al., 2014, 2017; Michaud et al., 2018).

The SML is also a direct recipient of atmospherically deposited aerosols, which have been shown to trigger community dissimilarities between SML and SSL bacterial populations in other regions, due to effects of nutrient inputs (Vila-Costa et al., 2013; Astrahan et al., 2016; Marín-Beltrán et al., 2019) and pollination events (Södergren, 1987) on bacterioneuston. The diffusive atmosphere-ocean exchange and snow deposition are the main sources of not only semivolatile OP, such as Polycyclic Aromatic Hydrocarbons (PAH) and polychlorinated biphenyls (Galbán-Malagón et al., 2013; Casal et al., 2018,2019), but also other hydrophobic and surfactant-like OPs such as perfluoroalkylsubstances (Casal et al., 2017). All these OP become part of the anthropogenic dissolved organic carbon (ADOC) (Vila-Costa et al., 2020) pool, which due to its hydrophobic and surfactant-like properties is enriched in the SML (Cincinelli et al., 2005; Stortini et al., 2009; Casas et al., 2020). The accumulation of ADOC and other surfactant-like organic matter is driven by rising bubbles with chemicals accumulated at the air-water interface and by ADOC attachment to buoyant particles from subsurface waters (Hunter, 2009). Removal processes of ADOC in the SML are volatilization and sea-spray aerosolization (Ehrenhauser et al., 2014), photodegradation (King et al., 2014; Yuan et al., 2019), settling of particulate matter, and microbial degradation (Coelho et al., 2011, 2013).

Ocean microbiomes harbor a subset of bacteria known to play a central role in the biodegradation of ADOC (Head et al., 2006; Berry and Gutierrez, 2017). Acute pollution events, as well as research with isolates, have revealed the genetic battery required for degradation of various families of ADOC constituents by specific taxa, as well as some of the degradation pathways mediated by these strains (Head et al., 2006; Takahashi et al., 2013; Dombrowski et al., 2016). A key microbial strategy that influences the bioavailability of ADOC is the bacterial production of biosurfactants, amphiphilic biomolecules encompassing a large spectrum of chemical families, that facilitate the solubilization and emulsification of ADOC compounds, mostly hydrophobic, or by changing the properties of the bacterial cell wall (Matvyeyeva et al., 2014; Andualem Bezza and Chirwa, 2015; Wittgens et al., 2017). The accumulation of amphiphilic and hydrophobic compounds in the SML modifies its surface tension, thus modifying the rate of air-water diffusive exchange of gases (Salter et al., 2009; Kurata et al., 2016) and atmospheric deposition of aerosols (Del Vento and Dachs, 2007). All these processes depict the SML as a highly dynamic system for interactions between organic matter and bacterioneuston (Wurl and Obbard, 2004), which has not yet been studied in depth, and never addressed for the Southern Ocean.

The goal of this study was to better understand the temporal dynamics and main environmental drivers of bacterial abundance and community composition in the SML in the maritime Antarctica. This was done by comparing, for the first time, SML and SSL community taxonomical structure during the nutrient-rich upwelling summer season in South Bay (Livingston Island, Antarctica). We characterized microbial communities by flow cytometry and 16S rRNA gene sequencing, and correlated the data with different water and air physicochemical variables, as well as perfluoroalkyl substances, taken as surrogates of hydrophobic and surfactant organic matter, as well as the SML stability.

## Materials and Methods

### Site Description and Sampling

**FIGURE 1:**
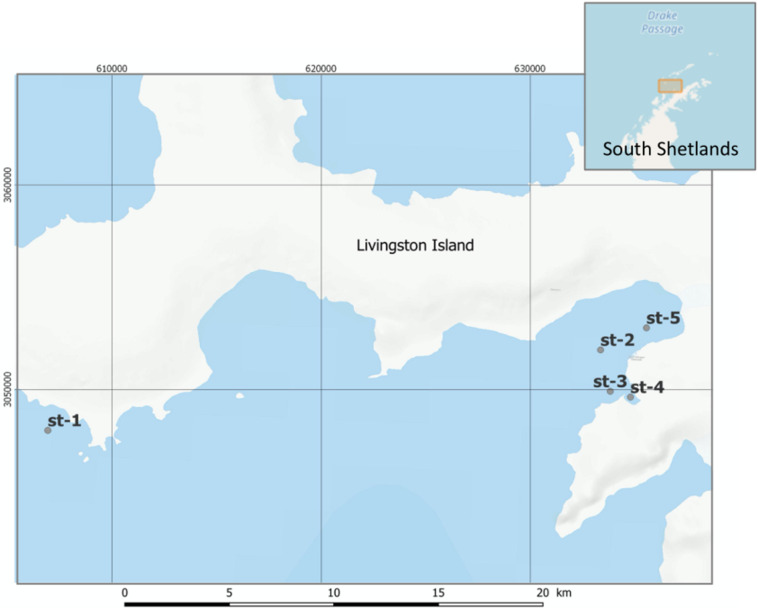
Location of the sampling stations for the SML and SSL at South Bay of Livingston Island (South Shetlands, Antarctica).

Samples were collected during austral summertime 2018, from January 8th to March 1st, at the South Bay of Livingston Island (South Shetland, Antarctica, 62° 39’ S, 60° 23’ W). SML and SSL sampling was conducted from a rigid inflatable boat at five different locations (Figure 1 and Supplementary Table S1). Physicochemical parameters were measured at each sampling station with a CTD probe. The SML was sampled with a glass plate SML sampler. This method was first described by Harvey and Burzell (1972) and has been used in a number of studies for sampling organic matter, microorganisms, surfactants, and organic pollutants related to SML investigations (Knulst et al., 2003; García-Flor et al., 2005; Ju et al., 2008). The pre rinsed plate (40 × 30 cm) was inserted vertically into the water, and then withdrawn slowly allowing the SML adhere onto the glass plate. The glass plate containing the SML sample was then wiped between two polypropylene (PP) plates draining the sample into PP bottles. This process was repeated until obtaining a volume of 1.5 L of SML water. Subsurface layer seawater samples were collected in 2 L PP bottles by submerging the bottles and opening it at 0.5 m depth.

### Meteorological Data

The meteorological parameters were provided by the Spanish Meteorological Agency (AEMET) and measured at the meteorological station at Livingston Island (62°39′46.2″S 60°23′24.6″W). The values were averaged coinciding, roughly, with the sampling hours (From 9 am to 11.30 am).

### Prokaryotic Cell Abundance

Subsamples of 1.8 mL for quantification of abundances of prokaryotes were fixed with 1% buffered paraformaldehyde solution (pH 7.0) plus 0.05% glutaraldehyde, left at room temperature in the dark for 10 min, and frozen and stored at −80°C until further processing. Prokaryotic cell abundance was estimated by flow cytometry as described elsewhere (Gasol and Morán, 2015).

### Perfluoroalkyl Compounds Extraction and Detection

SML and SSL samples were taken simultaneously to the samples used for bacterial analysis. Seawater was analyzed as reported previously (Casal et al., 2017). Briefly, samples were filtered through pre-combusted glass fiber filters (47 mm, GF/F Whatman). The analytes were extracted using solid phase extraction (SPE) with OASIS WAX cartridges (Waters). After loading 500 mL of SML water and 2 L of SSL water through the cartridges, these were stored at −20°C in sealed bags until their elution with methanol, followed by methanol containing 0.1% ammonia in an ultraclean laboratory. PFAS analysis was performed by Ultra Performance Liquid Chromatography tandem triple quadrupole mass spectrometry (UPLC-MS/MS) based on an established method with minor modifications (González-Gaya et al., 2014). Details of methods and results of PFAS in the SML and SSL are reported and discussed elsewhere (Casas et al., 2020) and used here when needed.

### Nutrients

Sub-samples (15 mL) were taken and kept at −20°C for analyzing dissolved inorganic nutrients, nitrate + nitrite (NO_–__3_ + NO_–__2_), ammonium (NH_4_^–^) and Phosphate (PO_4_^3–^). Analyses were done by standard segmented flow with colorimetric detection (Grasshoff et al., 1999) using a SEAL Analyzer AA3 HR. H_3_PO_4_ was added to acidify the sample to pH 2 and the ampoules were heat-sealed and stored in the dark at 4°C until analysis. Samples were measured with a SHIMADZU TOC-5000 analyzer, following the high-temperature catalytic oxidation (HTCO) technique (Cauwet, 1994). For the inorganic nutrients analysis, detection limits (defined as three times the standard deviation of 10 replicates at 50% diluted samples) were 0.006 μM for NO_–__3_, 0.003 μM for NO_–__2_, 0.003 μM for NH_4_^+^, and 0.01 μM for PO_4_^3–^.

### DNA Extraction, Library Preparation, and Taxonomical Identification of 16S rDNA Amplicons

Samples for 16S rDNA library construction were collected from SML and SSL. 400 mL of each sample were prefiltered through 3 μm pore-size 47 mm diameter polytetrafluoroethylene filters (Millipore, Billerica, MA) to remove grazers and the particle-attached living fraction, and sequentially onto 0.2 μm pore-size 47 mm PTFE (Millipore, Billerica, MA) under filters to capture the free-living bacteria cells fraction, using a peristaltic pump with a flow of <50 mL min^–1^.

Each filter was placed in 1 mL lysis buffer (50 mM Tris HCl, 40 mM EDTA, 0.75 M sucrose). All filters were stored at -20°C until further processing. After unthawing samples were incubated with lysozyme, proteinase K and sodium dodecyl sulfate (SDS), and nucleic acids were extracted simultaneously with phenol/chloroform/isoamyl alcohol (25: 24: 1 vol: vol: vol) and with chloroform/isoamyl alcohol (24: 1, vol: vol) as described in Vila-Costa et al. (2013). The resulting solution was concentrated to 200 μL using an Amicon Ultra 10-kDa filter unit (Millipore). Partial bacterial 16S gene fragments of both DNA were amplified using primers 515f/926r (Parada et al., 2016) plus adaptors for Illumina MiSeq sequencing. The PCR reaction mixture was thermocycled at 95°C for 3 min, 30 cycles at 95°C for 45 s, 50°C for 45 s, and 68°C for 90 s, followed by a final extension of 5 min at 68°C. PCR amplicon sizes were checked in tris- acetate-EDTA (TAE) agarose gels. Illumina MiSeq sequencing was conducted at the Pompeu Fabra University Sequencing Service.

### Bioinformatics

DADA2 v1.4 was used to differentiate the 16S V4-5 amplicon sequence variants (ASVs) and remove chimeras (parameters: maxN = 0, maxEE = 2.4, trunclen = 227,210; Callahan et al., 2016). DADA2 resolves ASVs by modeling the errors in Illumina-sequenced amplicon reads. The approach is threshold-free, inferring exact variants up to one nucleotide difference using the quality score distribution in a probability model. Previously, spurious sequences and primers were trimmed using cutadapt v.1.16 (Martin, 2011). Taxonomic assignment of the ASVs was performed with the RDP algorithm classifier against RDP database release 11.5 (Cole et al., 2014). ASVs classified as Mitochondria or Chloroplast were removed. The final ASV table contained 46 samples, obtaining for the entire sample set 549,256 amplicon sequence variants (ASV) from 16S rRNA gene V4-5 fragments, from which 12,769 were unique. Unique bacterial ASV averaged 291 and 267 per sample in SML and SSL, respectively.

### Statistical Analyses

Statistically significant differences between layers of abiotic and biotic parameters were assessed by Wilcoxon signed-rank test (*p* < 0.05) using the wilcox.test() function from “dplyr” package in R. Package “ggpubr” was used for spearman correlations significance (*p* ≤ 0.05) and the bivariate plots from the spearman significant correlations. Shannon Diversity indexes were calculated with diversity() function from “vegan v1.4-4” package. Further graphs were carried out using the “ggplot2” package, also in R environment (Wickham, 2009). Enrichment factors in the SML were calculated as the ratio between absolute values in the SML respect to the SSL. Absolute values were calculated multiplying the 16S relative values of each group by the absolute counts of total bacteria obtained by flow cytometry. Fold changes were calculated similarly using the 16S relative values with function foldchange() from “gtools” package (R Core Team, 2014).

## Results and Discussion

### Characterization of the Sampling Domain

The SML and SSL samples were collected during the Austral summer, from early January to early March in 2018, at five sampling sites of South Bay, Livingston Island (South Shetlands), representative of the maritime Antarctica (Figure 1 and Supplementary Table S1). Over the sampling period, sea surface temperature (SST) averaged 1.7 ± 0.4°C, and atmospheric temperature and solar radiation were 2.6 ± 1.07°C and 62.9 ± 47.46 Wm^–2^, respectively (Supplementary Tables S2, S3). Surface seawater from the three most coastal sites (St-3, St-4, and St-5), influenced by terrestrial inputs such as glacial meltdown runoff during the summer season, averaged 33.5 ± 0.2 PSU for salinity, 7.7 ± 2 FTU for turbidity and 1.6 ± 0.2°C for SST. These values were statistically different (*p* < 0.05, Student’s *t*-test) than those from the most off-shore sites (St-1 and St-2) which averaged in surface waters 34.0 ± 0.4 PSU for salinity, 4.4 ± 1.4 FTU for turbidity, and 2.1 ± 0.5°C for SST.

During summer months, strong upwelling events are caused by the Antarctic divergence and westerly winds inducing nutrient rich waters. Dissolved inorganic nitrogen and phosphate measures were within the range of those previously reported in Antarctic surface waters (<10 m) during summer months (Garcia et al., 2013), confirming a non-limited nutrient system. Averaged phosphate (PO_4_^3–^) concentrations showed a significant enrichment in the SML (1.7 ± 0.2 μmolL^–1^ at SML and 1.4 ± 0.3 μmolL^–1^ at SSL, *P* = 0.027, Paired Wilcoxon test). Conversely, Nitrate + nitrite (NO_–__3_ + NO_–__2_) concentrations in the SML were not significantly different than in the SSL (averaged 22 ± 4.1 μmolL^–1^ at SML and 19.1 ± 5.4 μmolL^–1^ at SSL), and ammonium (NH_4_^–^) showed a large variability across the dataset (averaged 4.3 ± 3.7 μmolL^–1^ at SML and 6.3 ± 7 μmolL^–1^ at SSL) (Supplementary Table S4 and Supplementary Figure S1).

The level of pollution by anthropogenic dissolved organic carbon was tracked by taking the concentrations of perfluoroalkyl substances (PFASs) as a proxy of ADOC. PFAS are organic synthetic compounds globally distributed by oceanic currents and by atmospheric transport through marine aerosols and deposition (Casal et al., 2017). PFAS were measured for the same set of samples than bacterioneuston (Casas et al., 2020). Thanks to their amphiphilic nature, PFAS have surfactant properties. In addition, PFAS have been shown to accumulate in planktonic organisms (Casal et al., 2017) due to their hydrophobicity. PFAS also accumulate in the SML (Casas et al., 2020). Surfactants have often been used as surrogates of the occurrence of the SML and its stability (Biffinger et al., 2004; Ju et al., 2008), thus we took PFAS as proxy of a developed SML enriched in ADOC. Specifically, we take the common perfluorooctaesulfonic acid (PFOS) as proxy of a surfactant and anthropogenic chemical. Recently, this compound has also been found to be partially degradable by Antarctic bacteria through desulfurization (Cerro-Gálvez et al., 2020). PFOS concentrations were significantly higher in the SML than in the SSL (50.3 ± 50.8 pgL^–1^ and 16.5 ± 8.7 pgL^–1^ respectively, paired Wilcoxon test, *p* = 0.009). The concentrations in the SSL are comparable to those reported for this region previously (Casal et al., 2017), and in some regions of the Atlantic, Pacific and Indian Oceans (González-Gaya et al., 2014). Enrichment factors (EF), defined as the ratio of PFOS concentration in the SML over the SSL, fluctuated from 1.2 up to 26.3 along the time series. PFOS concentration peaked on 27/01/2018 at 230 pgL^–1^ at the SML, indicating a very high stable event at the SML over the period 22/01/2018 – 03/02/2018, coinciding with a peak of SST (2.47–1.87°C), atmospheric temperature (2.7–5.6°C) and solar radiation (153.8–136.6 Wm^–2^) on the 27/01/2018 and 28/01/2018. Contrasting with these conditions, on 20/01/2018, 08/02/2018 and 16/02/2018 minor differences between PFOS concentration at both layers were found, consistent with disruption of the SML, possibly caused by wind speed (>5 ms^–1^), yielding low SML stability and a breakdown of surface stratification (Supplementary Table S4 and Supplementary Figure S1).

### Structure of Bacterioneuston and Bacterioplankton Communities

**FIGURE 2:**
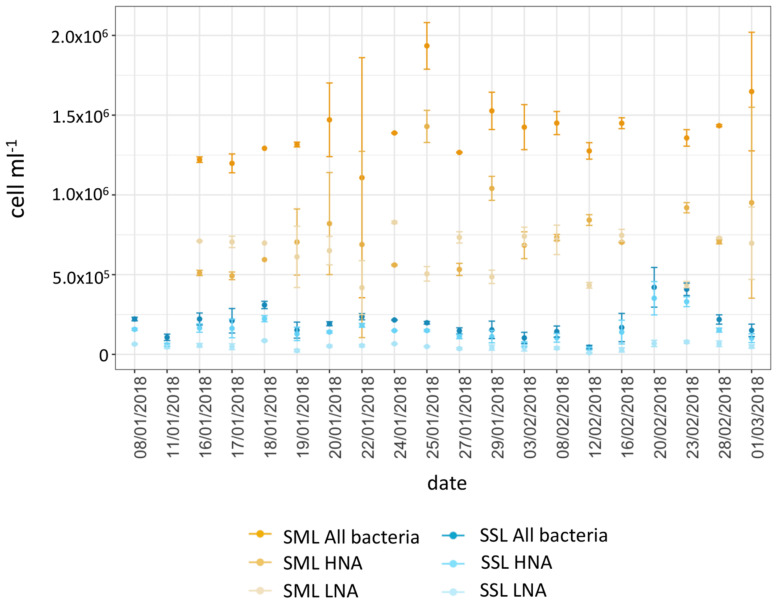
Bacterial abundance of LNA, HNA, and total heterotrophic bacteria for SML and SSL during the austral summer. Values are means of duplicates. Error bars show standard deviations.

Pairs of SML and SSL samples showed a significant higher bacterial abundance for bacterioneuston compared to bacterioplankton in all samples (Paired Wilcoxon test, *P* = 0.004), consistent with observations from other oceanic regions and freshwaters (Cunliffe et al., 2013). Enrichment factors averaged 9.1 ± 5.8 for total bacterial abundance. For high and low nucleic acid bacteria (HNA and LNA), the average abundances were 7.6 × 10^5^ ± 2.8 × 10^5^ cells ml^−1^ and 6.4 × 10^5^ ± 1.5 × 10^5^ cells ml^−1^ for SML, and 1.4 × 10^5^ ± 6.7 × 10^4^ cells ml^−1^ and 4.8 × 10^4^ ± 2 × 10^4^ cells ml^−1^ for SSL (Figure 2), respectively. Significant higher enrichment factors were observed for LNA cells compared to HNA cells, with an average 16.8 ± 10.6 for LNA, and 6.7 ± 5.1 for HNA. LNA bacteria, with smaller cell size and lower DNA content, have usually been regarded as the inactive fraction of the microbial community, with observed lower LNA specific growth rates and lower production of cell numbers in enrichment cultures compared to HNA (Wang et al., 2009; Vila-Costa et al., 2012). Higher LNA enrichment in the SML could be explained by the smaller cell size of this bacterial fraction, which becomes an advantageous trait for vertical transportation from subsurface waters and accumulation at the top surface layer (Michaud et al., 2018).

Significant differences of 16S amplicon sequence variants (ASVs) distribution were observed between sampling stations consistent with the pattern observed with physical and chemical parameters (Permanova test, *p* = 0.001, Supplementary Figure S2). While Layer (SML vs. SSL) appeared as a modest, yet significant, source of variability (*P* = 0.044), the interaction of both, sampling Station and Layer, accounted for a significant principal factor of variation (*P* = 0.001). Concerning the biodiversity, the Shannon index averaged 3.7 ± 0.7 at the SML, and 3.6 ± 0.6 at the SSL. Cell abundances were not significantly correlated to 16S diversity indices as it has been observed for the SML at other sites (Zäncker et al., 2018). This suggests a common core microbiome, enriched in the SML, and a limited number of taxa differentiating between layers and sites.

Bacterial assemblages were dominated by Flavobacteriia (30.5 ± 9.5% at SML and 30.1 ± 10.1% at SSL), Gammaproteobacteria (26.8 ± 15% at SML and 26.2 ± 16.6% at SSL), SAR11 clade (17.2 ± 7.9% at SML and 16.1 ± 9.2% at SSL) and Rhodobacterales (15.5 ± 8.1% at SML and 13.1 ± 5% at SSL) (Supplementary Figure S3). The bacterial composition of SSL was similar to those described previously in this region (Wilkins et al., 2013; Cavicchioli, 2015).

Both the SML and SSL microbiomes included genera with known facultative and obligate ADOC degraders (AD) strains, some of them also associated with biosurfactant production (see Supplementary Table S5 for details). Relative to the total bacterial community, AD averaged 12.3 ± 10.2% at SML and 10.3 ± 7.6% at SSL (for relative abundances of AD within their taxonomical group see Supplementary Figure S4).

**FIGURE 3:**
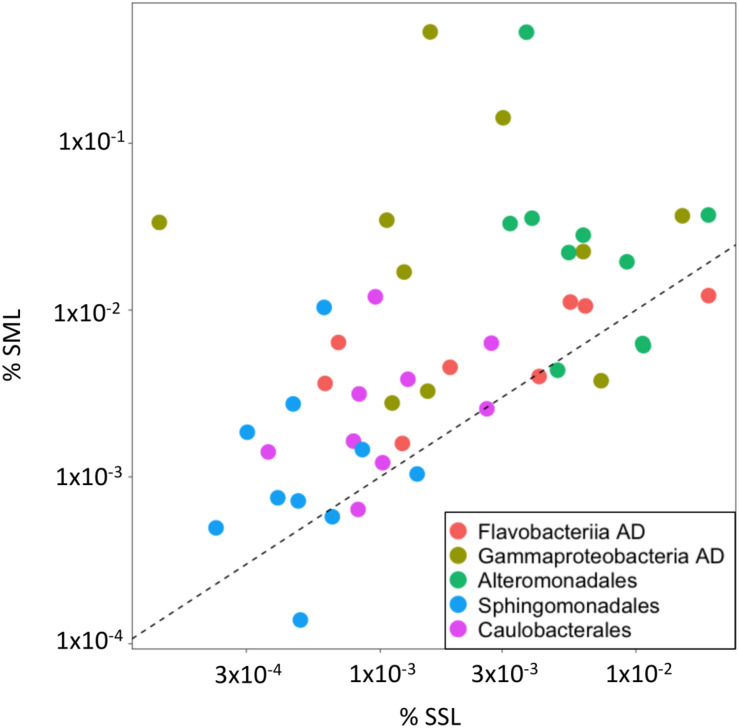
Relative abundances in the SML vs. SSL for selected taxa. Flavobactereiia AD and Gammaproteobacteria AD were significantly enriched in the SML, while Alteromonadales, Sphingomonadales and Caulobacterales presented high positive fold changes in the SML (FC > 2). Notice the logarithmic scale of the axes. Data points on the 1:1 dashed line indicate equal contribution to the 16S ASV pool.

**FIGURE 4:**
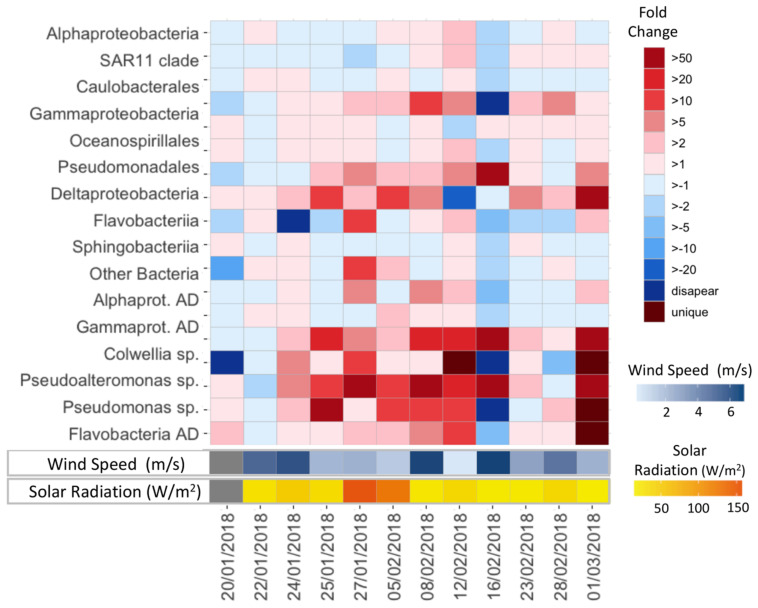
Fold change values in relative abundances of specific taxa in the SML vs. SSL during the austral summer.

At a finer taxonomic resolution, higher relative abundances of specific AD bacteria, known to be stimulated under high ADOC concentrations, were observed at the SML. The fold change of these groups in the SML vs. the SSL ranged from 1.5 to 50 (Figures 3, 4). Specifically, on average, significant higher relative abundances were observed for Gammaproteobacteria AD (*FC* = 10.26) and Flavobacteriia AD (*FC* = 1.68) (Wilcoxon paired data test *p* < 0.05). Other ADOC associated taxonomical groups with >2-fold increase in relative abundances at the SML for the overall data set were Alteromonadales (*FC* = 6.4), Caulobacterales (*FC* = 2.5), and Sphingomonadales (*FC* = 2.16) (Figure 3). Further, in a day-by-day basis, some gammaproteobacterial groups were systematically highly enriched at SML during the austral summer (Figure 4). Gammaproteobacteria AD and the ADOC-associated orders Alteromonadales and Pseudomonadales, taken separately, showed fold changes from >5 to >50 over various sampling days at the SML, and *Pseudoalteromonas*, *Colwellia*, *Pseudomonas* bloomed at the SML from 24th January onward. *Colwellia*, *Pseudomonas*, and *Pseudoalteromanas* are generalist strains with rapid facultative degrading capabilities toward ADOC, but also prompt to degrade other dissolved organic matter compounds. *Colwellia* have been described as abundant and fast responder following ADOC background concentration exposures in polar environments (Cerro-Gálvez et al., 2019), and to oil spills in marine environments (Gutierrez et al., 2013; Dombrowski et al., 2016; Lofthus et al., 2018; Vergeynst et al., 2018). In fact, *Colwellia* single cell assembled genomes (SAGs) retrieved from contaminated marine sites have revealed a more specific metabolic activity toward aromatic hydrocarbons, as well as a full set of genes involved with chemotaxis, motility and adaptations to cold environments such as Antarctica (Mason et al., 2014).

As some of these taxa are not obligate ADOC consumers, it is relevant to address whether the SML is enriched in dissolved organic carbon (DOC) or only in ADOC and biosurfactants. Previous works have shown that DOC in seawater from this region had values of 62 ± 7 μmol C L^–1^, while DOC concentrations in the SML were of 69 ± 11 μmol C L^–1^ (Calleja et al., 2009; Ruiz-Halpern et al., 2014). So the differences in concentrations are small in terms of bulk DOC. In Antarctica, several works have reported the enrichment of hydrophobic pollutants in the Southern Ocean SML, such as for n-alkanes, PAHs, PCBs, PFAS and phthalates (Cincinelli et al., 2005; Stortini et al., 2009; Casas et al., 2020). A recent work for the Arctic ocean (Chance et al., 2018), did find a specific enrichment of surfactant-like substances in the SML, but not of the total pool of DOC. These reports suggest that the moderate reported enrichment of DOC in the SML, when occurring, may be mostly driven by the fractions of hydrophobic and surfactant-like DOC, but probably not other pools of DOC.

### Environmental Drivers of Community Compositions. Can Anthropogenic and Surfactant-Like Organic Compounds Modulate Bacterioneuston Communities?

**FIGURE 5:**
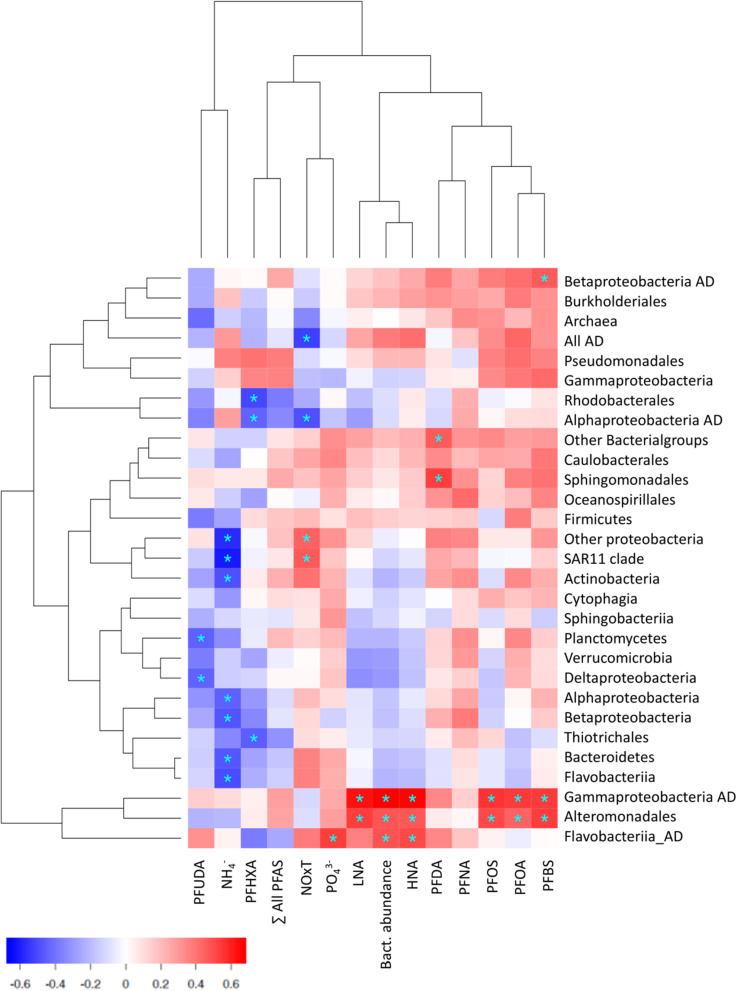
Correlation map between nutrients, PFAS and bacterial abundances and prevalence of bacterial groups. Red and blue sectors represent positive and negative correlations, asterisks indicate significant correlations (Spearman correlation, *p* ≤ 0.05). In the x axis: PFUDA, Perfluoroundecanoic acid; PFHXA, Perfluorohexanoic acid; PFDA, Perfluorodecanoic acid; PFNA, Perfluorononanoic acid; PFOS, Perfluorooctane sulfonate; PFOA, Perfluorooctanoic Acid; PFBS, Perfluorobutane sulfonate; LNA, Low Nucleic Acid; HNA, High Nucleic Acid.

Bacterioneuston community composition is controlled by physicochemical and biological parameters, including nutrient concentrations, phytoplankton assemblages and their exudates (Taylor et al., 2014). Bivariate correlations between environmental and bacterial composition variables, considering all measures in the SML and SSL, show that phosphate concentrations were positively correlated to most of the taxonomical groups, and significantly to specific Flavobacteriia AD, while ammonia concentrations were negatively correlated to Bacteroidetes phyla, Flavobacteriia, Alphaproteobacteria, SAR11 Clade and Actinobacteria, pointing to the pivotal role of nutrients as key drivers of microbial compositions (Figure 5; Allen et al., 2012; Cavicchioli, 2015). Further, relative abundances of Alteromonadales and Gammaproteobacteria AD were positively correlated to concentrations of PFOS and other PFAS (Spearman correlation, *p* ≤ 0.05, Figure 5). Also, Perfluorohexanoic acid (PFHXA) correlated negatively to dominant Rhodobacterales and alphaproteobacterial AD, suggesting toxic consequences of part of ADOC toward these taxa. These correlations are consistent with ADOC modulating the structure of community composition and is aligns with the dual effect of ADOC compounds on marine microorganisms (Head et al., 2006; Rodríguez et al., 2018). From one side, ADOC can be a source of nutrients and organic carbon, and on the contrary, ADOC compounds can induce toxicity to bacteria (see e.g., Cerro-Gálvez et al., 2019, 2020; Vila-Costa et al., 2019). Our observations are consistent with previously documented microbial responses triggered by ADOC pollution events and in chronically polluted marine environments (Head et al., 2006; Hazen et al., 2010; Rivers et al., 2013; Joye et al., 2016).

The specific SML highly enriched taxa, Gammaproteobacteria AD and Flavobacteriia AD, but also ADOC-associated orders such as Alteromonadales and Pseudomonadales, coincide with those taxonomical groups not only known to be capable of degrading ADOC (such as hydrocarbons) or that have been observed to be stimulated by the presence of oil (Hazen et al., 2010; Neethu et al., 2019) but also with a described resistance to oxidative stress situations such as those originated by UV radiation (Alonso-Sáez et al., 2006; Ruiz-González et al., 2013; Svenningsen et al., 2015). The presence of ROS-resistant ADOC-degrading bacterioneuston have been reported in other studies in aquatic environments, proving ADOC metabolism at the SML (Guerin, 1989; Coelho et al., 2011). To our knowledge, this is the first report of their occurrence in polar regions.

**FIGURE 6:**
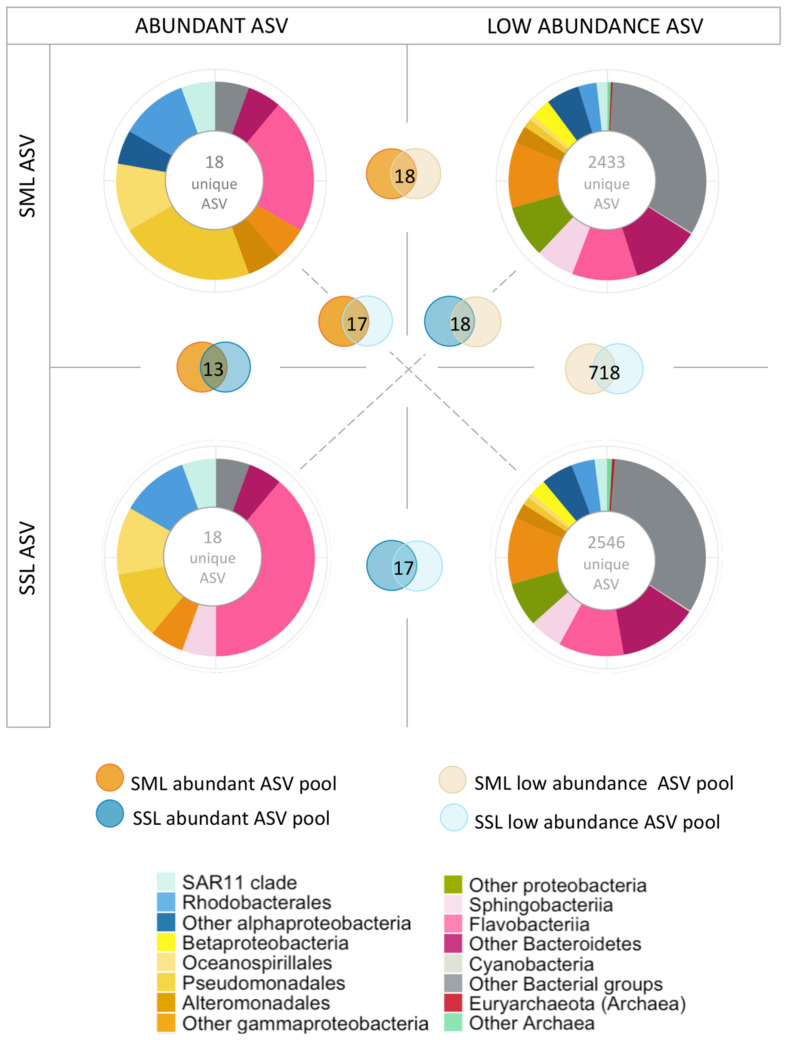
Low abundance and abundant taxa analysis based on 16S rDNA ASV counts. Taxonomical distribution of main taxonomical groups assigned to ASVs with high abundances (>3%) and low abundances (<3%). Overlapping circles indicate the number of unique ASVs shared in the different abundance pools.

The consistently significant higher enrichment of taxon-specific ADOC degraders at the SML than other taxa in a day-by-day basis coincides with the taxa found at low relative abundances (<3%) in the underlying SSL (examples of these are: on the 25/01/2018 *Pseudomonas* accounted for 6.3% at SML and 0.1% at the SSL; on the 16/02/2018 *Pseudoalteromonas* accounted for 46.2% at SML and 0.03% at SSL, among others). This is consistent with the observed growth of ADOC stimulated rare biosphere after oil spills and under ADOC exposure at environmental levels (Wang et al., 2017; Cerro-Gálvez et al., 2019). This large repository of dormant and low abundance bacterial taxa are part of a local seed bank which specially benefits from ecosystem variations such as sudden growth substrate availability or environmental advantageous conditions (Galand and Logares, 2019). The large temporal and spatial variability of the SML may favor the occasional or opportunistic growth of these taxa. In our data set, low abundant taxa (<3%) represented a pool of 2433 and 2546 unique ASVs at the SML and SSL respectively, with 718 ASV shared between both low abundance ASV pools (Figure 6). On the other side, abundant ASVs pools included 18 unique ASV for both layers, and all of them were also present at the low abundance ASV pools. These results point to a rapid growth of opportunist or specialist species at the SML, possibly recruited and transported vertically from the underlying bulk water low abundance biosphere, and with the required resistance traits or fast UV-repair mechanism to withstand SML extreme environmental conditions.

Many of the taxa described as ADOC degraders have also been reported to be biosurfactant producers (Supplementary Table S5). Biosurfactants play a role by both promoting ADOC degradation and their detoxification. On the other hand, their amphiphilic properties favor their accumulation in the surface microlayer. The observation of large enrichments of ADOC degrading taxa may lead to a dynamic synergistic interaction between bacteria, pollutants and surfactants. The latter may also have contributions from other planktonic groups (Ẑutic et al., 1981; Wurl et al., 2009). All these processes may show a synergy favoring the fast turnover of ADOC in the marine environment, and future work is required to elucidate its relevance.

## Conclusion

We analyzed the bacterial community composition and abundance in paired SML and SSL samples over the austral summer in coastal seawater of Livingston Island (South Shetlands, Antarctica), together with chemical and physical measurements. Whereas total bacterial abundances were higher in the SML than in the SSL, both layers shared a similar taxa composition, which varied spatially among sampling sites. The main significant differences between SML and SSL samples from the same site corresponded to higher relative abundances at the SML of taxa reported to be ADOC degraders, such as Gammaproteobacteria AD and Flavobacteriia AD, and ADOC-associated orders Alteromonadales, Caulobacterales and Sphingomonadales. These taxa showed remarkably higher SML enrichments than the rest of the microbiome. In a day-by-day analysis, we observed that specific gammaproteobacteria taxa were systematically enriched at the SML, while the same taxa were found at low relative abundances in the underlying bulk water, supporting the hypothesis that bacterioneuston composition might be directly sourced from differing adjacent environments indigenous bacterial communities belonging to a low abundant or even rare biosphere. We found significant positive correlations between the relative abundances of Alteromonadales, Gammaproteobacteria AD and Sphinogmonadales and the concentrations of PFOS, used as a proxy of hydrophobic and surfactant-like ADOC. These results suggested that ADOC is a vector modulating bacterial compositions in the SML. While UV radiation, wind speed and organic matter have been proposed as the main influences determining bacterioneuston community composition (Agogué et al., 2005; Stolle et al., 2011; Rahlff et al., 2019), our findings provide field evidence that ADOC accumulation at the SML could also be another, although neglected, community composition driver. ADOC, as a double-edged sword, can become potential growth substrate for microorganisms, but also act as a toxic agent and hamper bacterial growth of other taxa. Our results suggest that, due to its unique interphase position, SML will respond sensitively to an increasing arrival and accumulation of anthropogenic organic pollution to the oceans. While bacterioneuston plays a central role in mediating biogeochemical processes at global scale, chronical ADOC pollution SML enrichments might modify the bacterioneuston’s bioreactor functioning, with a relevant biogeochemical role in the surface ocean.

## Data Availability Statement

The datasets generated for this study can be found in the online repositories. The names of the repository/repositories and accession number(s) can be found at: https://www.ncbi.nlm.nih.gov/, PRJNA637489; https://www.ncbi.nlm.nih.gov/, PRJNA648053.

## Author Contributions

AM-V, MV-C, and JD designed the sampling strategy and wrote the manuscript. AM-V, GC, and JD conducted the field sampling. AM-V performed the molecular work and data analyses. GC quantified the PFAS concentrations. BP performed the statistical analyses. All authors discussed the results and implications and commented on the final version of the manuscript.

## Conflict of Interest

The authors declare that the research was conducted in the absence of any commercial or financial relationships that could be construed as a potential conflict of interest.

## References

[B1] Agogué H. Casamayor E. O. Bourrain M. Obernosterer I. Joux F. Herndl G. J. (2005). A survey on bacteria inhabiting the sea surface microlayer of coastal ecosystems. *FEMS Microbiol. Ecol.* 54 269–280. 10.1016/j.femsec.2005.04.002 16332325

[B2] Allen L. Z. Allen E. E. Badger J. H. McCrow J. P. Paulsen I. T. Elbourne L. D. (2012). Influence of nutrients and currents on the genomic composition of microbes across an upwelling mosaic. *ISME J.* 6 1403–1414. 10.1038/ismej.2011.201 22278668 PMC3379637

[B3] Aller J. Y. Kuznetsova M. R. Jahns C. J. Kemp P. F. (2005). The sea surface microlayer as a source of viral and bacterial enrichment in marine aerosols. *J. Aerosol Sci.* 36 801–812. 10.1016/j.jaerosci.2004.10.012

[B4] Alonso-Sáez L. Gasol J. M. Lefort T. Hofer J. Sommaruga R. (2006). Effect of natural sunlight on bacterial activity and differential sensitivity of natural bacterioplankton groups in Northwestern Mediterranean coastal waters. *Appl. Environ. Microbiol.* 72 5806–5813. 10.1128/aem.00597-06 16957198 PMC1563624

[B5] Andualem Bezza F. Chirwa E. M. N. (2015). Biosurfactant-enhanced bioremediation of aged polycyclic aromatic hydrocarbons (PAHs) in creosote contaminated soil. *Chemosphere* 144 635–644. 10.1016/j.chemosphere.2015.08.027 26408261

[B6] Astrahan P. Herut B. Paytan A. Rahav E. (2016). The impact of dry atmospheric deposition on the sea-surface microlayer in the SE Mediterranean sea: an experimental approach. *Front. Mar. Sci.* 3:222 10.3389/fmars.2016.00222

[B7] Berry D. Gutierrez T. (2017). Evaluating the detection of hydrocarbon-degrading bacteria in 16S rRNA gene sequencing surveys. *Front. Microbiol.* 8:896. 10.3389/fmicb.2017.00896 PMC5434106 28567035

[B8] Biffinger J. C. Kim H. W. DiMagno S. G. (2004). The polar hydrophobicity of fluorinated compounds. *Chembiochem* 5 622–627. 10.1002/cbic.200300910 15122633

[B9] Brooks I. M. Yelland M. J. Upstill-Goddard R. C. Nightingale P. D. Archer S. d’Asaro E. (2009). Supplement to physical exchanges at the air-sea interface: UK-SOLAS field measurements. *Bull. Am. Meteorol. Soc.* 90 629–644.

[B10] Callahan B. J. McMurdie P. J. Rosen M. J. Han A. W. Johnson A. J. A. Holmes S. P. (2016). DADA2: high-resolution sample inference from Illumina amplicon data. *Nat. Methods* 13 581–583. 10.1038/nmeth.3869 27214047 PMC4927377

[B11] Calleja M. L. Duarte C. M. Prairie Y. T. Agustí S. Herndl G. J. (2009). Evidence for surface organic matter modulation of air-sea CO2 gas exchange. *Biogeosciences* 6 1105–1114. 10.5194/bg-6-1105-2009

[B12] Casal P. Cabrerizo A. Vila-Costa M. Jiménez B. Dachs J. (2018). Pivotal role of snow deposition and melting driving fluxes of polycyclic aromatic hydrocarbons at coastal livingston island (Antarctica). *Environ. Sci. Technol.* 52, 12327–12337. 30277758 10.1021/acs.est.8b03640

[B13] Casal P. Casas G. Vila-Costa M. Cabrerizo A. Pizarro M. Jiménez B. (2019). Snow amplification of persistent organic pollutants at coastal Antarctica. *Environ. Sci. Technol.* 53 8872–8882. 10.1021/acs.est.9b03006 31298532

[B14] Casal P. Zhang Y. Martin J. W. Pizarro M. Jiménez B. Dachs J. (2017). Role of snow deposition of perfluoroalkylated substances at coastal Livingston Island (Maritime Antarctica). *Environ. Sci. Technol.* 51 8460–8470. 10.1021/acs.est.7b02521 28665121

[B15] Casas G. Martinez-Varela A. Roscales J. L. Vila-Costa M. Dachs J. Jimenez B. (2020). Enrichment of perfluoroalkyl substances in the surface microlayer and sea-spray aerosols in the Southern Ocean. *Environ. Pollut*. 10.1016/j.envpol.2020.115512 32892018

[B16] Cavicchioli R. (2015). Microbial ecology of Antarctic aquatic systems. *Nat. Rev. Microbiol.* 13 691–706. 26456925 10.1038/nrmicro3549

[B17] Cauwet G. (1994). HTCO method for dissolved organic carbon analysis in seawater: influence of catalyst on blank estimation. *Mar. Chem.* 47, 55–64.

[B18] Cerro-Gálvez E. Casal P. Lundin D. Piña B. Pinhassi J. Dachs J. (2019). Microbial responses to anthropogenic dissolved organic carbon in the Arctic and Antarctic coastal seawaters. *Environ. Microbiol.* 21 1466–1481. 10.1111/1462-2920.14580 30838733

[B19] Cerro-Gálvez E. Roscales J. L. Jiménez B. Sala M. M. Dachs J. Vila-Costa M. (2020). Microbial responses to perfluoroalkyl substances and perfluorooctanesulfonate (PFOS) desulfurization in the Antarctic marine environment. *Water Res.* 171:115434. 10.1016/j.watres.2019.115434 31927092

[B20] Chance R. J. Hamilton J. F. Carpenter L. J. Hackenberg S. C. Andrews S. J. Wilson T. W. (2018). Water-soluble organic composition of the Arctic Sea surface microlayer and association with ice nucleation ability. *Environ. Sci. Technol.* 52 1817–1826. 10.1021/acs.est.7b04072 29370515

[B21] Cincinelli A. Stortini A. M. Checchini L. Martellini T. Del Bubba M. Lepri L. (2005). Enrichment of organic pollutants in the sea surface microlayer (SML) at Terra Nova Bay, Antarctica: influence of SML on superficial snow composition. *J. Environ. Monit.* 7 1305–1312. 10.1039/b507321a 16307088

[B22] Coelho F. Sousa S. Santos L. Santos A. Almeida A. Gomes N. (2011). Exploring hydrocarbonoclastic bacterial communities in the estuarine surface microlayer. *Aquat. Microb. Ecol.* 64 185–195. 10.3354/ame01526

[B23] Coelho F. J. R. C. Santos A. L. Coimbra J. Almeida A. Cunha Â. Cleary D. F. R. (2013). Interactive effects of global climate change and pollution on marine microbes: the way ahead. *Ecol. Evol.* 3 1808–1818. 10.1002/ece3.565 23789087 PMC3686211

[B24] Cole J. R. Wang Q. Fish J. A. Chai B. Mcgarrell D. M. Sun Y. (2014). Ribosomal Database Project: data and tools for high throughput rRNA analysis. *Nucleic Acids Res.* 42 D633–D642. 24288368 10.1093/nar/gkt1244 PMC3965039

[B25] Cunliffe M. Engel A. Frka S. Gašparoviæ B. Ž. Guitart C. Murrell J. C. (2013). Sea surface microlayers: a unified physicochemical and biological perspective of the air-ocean interface. *Prog. Oceanogr.* 109 104–116. 10.1016/j.pocean.2012.08.004

[B26] Del Vento S. Dachs J. (2007). Influence of the surface microlayer on atmospheric deposition of aerosols and polycyclic aromatic hydrocarbons. *Atmos. Environ.* 41 4920–4930. 10.1016/j.atmosenv.2007.01.062

[B27] Dombrowski N. Donaho J. A. Gutierrez T. Seitz K. W. Teske A. P. Baker B. J. (2016). Reconstructing metabolic pathways of hydrocarbon-degrading bacteria from the Deepwater Horizon oil spill. *Nat. Microbiol.* 1:16057. 10.1038/nmicrobiol.2016.57 27572965

[B28] Ehrenhauser F. S. Avij P. Shu X. Dugas V. Woodson I. Liyana-Arachchi T. (2014). Bubble bursting as an aerosol generation mechanism during an oil spill in the deep-sea environment: laboratory experimental demonstration of the transport pathway. *Environ. Sci. Process. Impacts* 16 65–73. 10.1039/c3em00390f 24296745

[B29] Engel A. Sperling M. Sun C. Grosse J. Friedrichs G. (2018). Organic matter in the surface microlayer: insights from a wind wave channel experiment. *Front. Mar. Sci.* 5:182 10.3389/fmars.2018.00182

[B30] Fan J. Li H. Chang Y. Wang X. Ming H. Su J. (2018). Spatial analysis of bacterioneuston and bacterioplankton diversity in the north China Sea. *Aquat. Ecosyst. Health Manag.* 21 1–9. 10.1080/14634988.2017.1400360

[B31] Galand P. E. Logares R. (2019). “Ecology of rare microorganisms,” in *Encyclopedia of Microbiology*, ed. Schmidt T. (Amsterdam: Elsevier), 90–96.

[B32] Galbán-Malagón C. J. Del Vento S. Berrojalbiz N. Ojeda M.-J. Dachs J. (2013). Polychlorinated biphenyls, hexachlorocyclohexanes and hexachlorobenzene in seawater and phytoplankton from the Southern Ocean (Weddell, South Scotia, and Bellingshausen Seas). *Environ. Sci. Technol.* 47 5578–5587. 10.1021/es400030q 23627767

[B33] Garbe C. Rutgersson A. Boutin J. Leeuw G. Delille B. (2014). “Transfer across the air-sea interface,” in *Ocean-Atmosphere Interactions of Gases and Particles*, eds Liss P. Johnson M. (Berlin: Springer), 55–112.

[B34] Garcia H. E. Locarnini R. A. Boyer T. P. Antonov J. I. Baranova O. K. Zweng M. M. (2013). “Dissolved inorganic nutrients (phosphate, nitrate, silicate),” in *World Ocean Atlas 2013*, Vol. 76 eds Levitus S. Mishonov A. (Silver Spring, MD: NESDIS), 25.

[B35] García-Flor N. Guitart C. Ábalos M. Dachs J. Bayona J. M. Albaigés J. (2005). Enrichment of organochlorine contaminants in the sea surface microlayer: an organic carbon-driven process. *Mar. Chem.* 96 331–345. 10.1016/j.marchem.2005.01.005

[B36] Gasol J. M. Morán X. A. G. (2015). “Flow cytometric determination of microbial abundances and its use to obtain indices of community structure and relative activity,” in *Hydrocarbon and Lipid Microbiology Protocols*, eds McGenity T. Timmis K. Nogales B. (Berlin: Springer), 159–187. 10.1007/8623_2015_139

[B37] González-Gaya B. Dachs J. Roscales J. L. Caballero G. Jiménez B. (2014). Perfluoroalkylated substances in the global tropical and subtropical surface oceans. *Environ. Sci. Technol.* 48 13076–13084. 10.1021/es503490z 25325411

[B38] Grasshoff K. Kremling K. Ehrhardt M. (1999). *Methods of Seawater Analysis*, 3rd Edn Weinheim: Wiley-VCH.

[B39] Guerin W. F. (1989). Phenanthrene degradation by estuarine surface microlayer and bulk water microbial populations. *Microb. Ecol.* 17 89–104. 10.1007/bf02025596 24197126

[B40] Gutierrez T. Singleton D. R. Berry D. Yang T. Aitken M. D. Teske A. (2013). Hydrocarbon-degrading bacteria enriched by the Deepwater Horizon oil spill identified by cultivation and DNA-SIP. *ISME J.* 7 2091–2104. 10.1038/ismej.2013.98 23788333 PMC3806270

[B41] Harvey G. W. Burzell L. A. (1972). A simple microlayer method for small samples. *Limnol. Oceanogr.* 17 156–157. 10.4319/lo.1972.17.1.0156

[B42] Hazen T. C. Dubinsky E. A. Desantis T. Z. Andersen G. L. Piceno Y. M. Singh N. (2010). Deep-sea oil plume enriches indigenous oil-degrading bacteria. *Science* 330 204–208. 10.1126/science.1195979 20736401

[B43] Head I. M. Jones D. M. Röling W. F. M. (2006). Marine microorganisms make a meal of oil. *Nat. Rev. Microbiol.* 4 173–182. 10.1038/nrmicro1348 16489346

[B44] Hervas A. Casamayor E. O. (2009). High similarity between bacterioneuston and airborne bacterial community compositions in a high mountain lake area. *FEMS Microbiol. Ecol.* 67 219–228. 10.1111/j.1574-6941.2008.00617.x 19049500

[B45] Hunter K. A. (2009). “Chemistry of the sea-surface microlayer,” in *The Sea Surface and Global Change*, eds Liss P. S. Duce R. A. (Cambridge: Cambridge University Press), 287–320. 10.1017/cbo9780511525025.010

[B46] Joye S. Kleindienst S. Gilbert J. Handley K. Weisenhorn P. Overholt W. (2016). Responses of microbial communities to hydrocarbon exposures. *Oceanography* 29 136–149. 10.5670/oceanog.2016.78

[B47] Ju X. Jin Y. Sasaki K. Saito N. (2008). Perfluorinated surfactants in surface, subsurface water and microlayer from dalian coastal waters in China. *Environ. Sci. Technol.* 42 3538–3542. 10.1021/es703006d 18546686

[B48] King S. M. Leaf P. A. Olson A. C. Ray P. Z. Tarr M. A. (2014). Photolytic and photocatalytic degradation of surface oil from the Deepwater Horizon spill. *Chemosphere* 95 415–422. 10.1016/j.chemosphere.2013.09.060 24139429

[B49] Knulst J. C. Rosenberger D. Thompson B. Paatero J. (2003). Intensive sea surface microlayer investigations of open leads in the pack ice during Arctic Ocean 2001 expedition. *Langmuir* 19 10194–10199. 10.1021/la035069

[B50] Kurata N. Vella K. Hamilton B. Shivji M. Soloviev A. Matt S. (2016). Surfactant-associated bacteria in the near-surface layer of the ocean. *Sci. Rep.* 6:19123. 10.1038/srep19123 PMC4709576 26753514

[B51] Kuznetsova M. Lee C. (2001). Enhanced extracellular enzymatic peptide hydrolysis in the sea-surface microlayer. *Mar. Chem.* 73 319–332. 10.1016/s0304-4203(00)00116-x

[B52] Lofthus S. Netzer R. Lewin A. S. Heggeset T. M. B. Haugen T. Brakstad O. G. (2018). Biodegradation of n-alkanes on oil–seawater interfaces at different temperatures and microbial communities associated with the degradation. *Biodegradation* 29 141–157. 10.1007/s10532-018-9819-z 29397457

[B53] Marín-Beltrán I. Logue J. B. Andersson A. F. Peters F. (2019). Atmospheric deposition impact on bacterial community composition in the NW Mediterranea. *Front. Microbiol.* 10:858. 10.3389/fmicb.2019.00858 31068921 PMC6491866

[B54] Martin M. (2011). Cutadapt removes adapter sequences from high-throughput sequencing reads. *EMBnet J.* 17:10 10.14806/ej.17.1.200

[B55] Mason O. U. Han J. Woyke T. Jansson J. K. (2014). Single-cell genomics reveals features of a *Colwellia* species that was dominant during the Deepwater Horizon oil spill. *Front. Microbiol.* 5:332. 10.3389/fmicb.2014.00332 25071745 PMC4085564

[B56] Matvyeyeva O. L. Vasylchenko Î. À. Aliievà O. R. (2014). Microbial biosurfactants role in oil products biodegradation. *Int. J. Environ. Bioremediat. Biodegrad.* 2 69–74.

[B57] Mayol E. Arrieta J. M. Jiménez M. A. Martínez-Asensio A. Garcias-Bonet N. Dachs J. (2017). Long-range transport of airborne microbes over the global tropical and subtropical ocean. *Nat. Commun.* 8:201. 10.1038/s41467-017-00110-9 PMC5544686 28779070

[B58] Mayol E. Jiménez M. A. Herndl G. J. Duarte C. M. Arrieta J. M. (2014). Resolving the abundance and air- sea fluxes of airborne microorganisms in the North Atlantic Ocean. *Front. Microbiol.* 5:557. 10.3389/fmicb.2014.00557 25400625 PMC4215616

[B59] Michaud J. M. Thompson L. R. Kaul D. Espinoza J. L. Richter R. A. Xu Z. Z. (2018). Taxon-specific aerosolization of bacteria and viruses in an experimental ocean-atmosphere mesocosm. *Nat. Commun.* 9:2017. 10.1038/s41467-018-04409-z PMC5964107 29789621

[B60] Nakajima R. Tsuchiya K. Nakatomi N. Yoshida T. Tada Y. Konno F. (2013). Enrichment of microbial abundance in the sea-surface microlayer over a coral reef: implications for biogeochemical cycles in reef ecosystems. *Mar. Ecol. Prog. Ser.* 490 11–22.

[B61] Naumann E. (1917). Beiträge zur kenntnis des teichnannoplanktons, I. I. Über das neuston des süsswassers. *Biol. Cent* 37 98–106.

[B62] Neethu C. S. Saravanakumar C. Purvaja R. Robin R. S. Ramesh R. (2019). Oil-spill triggered shift in indigenous microbial structure and functional dynamics in different marine environmental matrices. *Sci. Rep.* 9:1354. 10.1038/s41598-018-37903-x PMC6361881 30718727

[B63] Obernosterer I. Catala P. Reinthaler T. Herndl G. J. Lebaron P. (2005). Enhanced heterotrophic activity in the surface microlayer of the Mediterranean Sea. *Aquat. Microb. Ecol.* 39 293–302.

[B64] Parada A. E. Needham D. M. Fuhrman J. A. (2016). Every base matters: assessing small subunit rRNA primers for marine microbiomes with mock communities, time series and global field samples. *Environ. Microbiol.* 18 1403–1414. 26271760 10.1111/1462-2920.13023

[B65] Parks G. Dean C. W. Kluge J. A. Soloviev A. V. Shivji M. Tartar A. (2020). Analysis of surfactant-associated bacteria in the sea surface microlayer using deoxyribonucleic acid sequencing and synthetic aperture radar. *Int. J. Remote Sens.* 41 3886–3901.

[B66] R Core Team (2014). *R: A Language and Environment for Statistical Computing.*. Vienna: R Foundation for Statistical Computing

[B67] Rahlff J. Stolle C. Giebel H.-A. Ribas-Ribas M. Damgaard L. R. Wurl O. (2019). Oxygen profiles across the sea-surface microlayer—effects of diffusion and biological activity. *Front. Mar. Sci.* 6:11 10.3389/fmars.2019.00011

[B68] Rivers A. R. Sharma S. Tringe S. G. Martin J. Joye S. B. Moran M. A. (2013). Transcriptional response of bathypelagic marine bacterioplankton to the Deepwater Horizon oil spill. *ISME J.* 7 2315–2329. 23902988 10.1038/ismej.2013.129 PMC3834857

[B69] Rodríguez J. Gallampois C. M. J. Timonen S. Andersson A. Sinkko H. Haglund P. (2018). Effects of organic pollutants on bacterial communities under future climate change scenarios. *Front. Microbiol.* 9:2926. 10.3389/fmicb.2018.02926 30555447 PMC6284067

[B70] Ruiz-González C. Simó R. Sommaruga R. Gasol J. M. (2013). Away from darkness: a review on the effects of solar radiation on heterotrophic bacterioplankton activity. *Front. Microbiol.* 4:131. 10.3389/fmicb.2013.00131 23734148 PMC3661993

[B71] Ruiz-Halpern S. Ll Calleja M. Dachs J. Del Vento S. Pastor M. Palmer M. (2014). Ocean-atmosphere exchange of organic carbon and CO 2 surrounding the Antarctic Peninsula. *Biogeosciences* 11 2755–2770.

[B72] Sabbaghzadeh B. Upstill-Goddard R. C. Beale R. Pereira R. Nightingale P. D. (2017). The Atlantic Ocean surface microlayer from 50°N to 50°S is ubiquitously enriched in surfactants at wind speeds up to 13 m s-1. *Geophys. Res. Lett.* 44 2852–2858.

[B73] Salter I. Zubkov M. V. Warwick P. E. Burkill P. H. (2009). Marine bacterioplankton can increase evaporation and gas transfer by metabolizing insoluble surfactants from the air-seawater interface. *FEMS Microbiol. Lett.* 294 225–231. 19431239 10.1111/j.1574-6968.2009.01572.x

[B74] Södergren A. (1987). Origin and composition of surface slicks in lakes of differing trophic status1. *Limnol. Oceanogr.* 32 1307–1316.

[B75] Stolle C. Labrenz M. Meeske C. Jürgens K. (2011). Bacterioneuston community structure in the southern Baltic sea and its dependence on meteorological conditions. *Appl. Environ. Microbiol.* 77 3726–3733. 21478321 10.1128/AEM.00042-11 PMC3127628

[B76] Stolle C. Nagel K. Labrenz M. Jürgens K. (2010). Bacterial activity in the sea-surface microlayer: in situ investigations in the Baltic Sea and the influence of sampling devices. *Aquat. Microb. Ecol.* 58 67–78.

[B77] Stortini A. M. Martellini T. Del Bubba M. Lepri L. Capodaglio G. Cincinelli A. (2009). n-Alkanes, PAHs and surfactants in the sea surface microlayer and sea water samples of the Gerlache Inlet sea (Antarctica). *Microchem. J.* 92 37–43.

[B78] Svenningsen N. B. Pérez-Pantoja D. Nikel P. I. Nicolaisen M. H. De Lorenzo V. Nybroe O. (2015). *Pseudomonas putida* mt-2 tolerates reactive oxygen species generated during matric stress by inducing a major oxidative defense response. *BMC Microbiol.* 15:202. 10.1186/s12866-015-0542-1 26445482 PMC4595014

[B79] Takahashi S. Abe K. Ker Y. (2013). “Microbial degradation of persistent organophosphorus flame retardants,” in *Environmental Biotechnology - New Approaches and Prospective Applications*, ed. Petre M. (London: InTech), 91.

[B80] Taylor J. D. Cottingham S. D. Billinge J. Cunliffe M. (2014). Seasonal microbial community dynamics correlate with phytoplankton-derived polysaccharides in surface coastal waters. *ISME J.* 8 245–248. 24132076 10.1038/ismej.2013.178 PMC3869024

[B81] Vergeynst L. Wegeberg S. Aamand J. Lassen P. Gosewinkel U. Fritt-Rasmussen J. (2018). Biodegradation of marine oil spills in the Arctic with a Greenland perspective. *Sci. Total Environ.* 626 1243–1258. 29898532 10.1016/j.scitotenv.2018.01.173

[B82] Vila-Costa M. Barberan A. Auguet J.-C. Sharma S. Moran M. A. Casamayor E. O. (2013). Bacterial and archaeal community structure in the surface microlayer of high mountain lakes examined under two atmospheric aerosol loading scenarios. *FEMS Microbiol. Ecol.* 84 387–397. 23289422 10.1111/1574-6941.12068

[B83] Vila-Costa M. Cerro-Galvez E. Martinez-Varela A. Casas G. Dachs J. (2020). Anthropogenic dissolved organic carbon and marine microbiomes. *ISME J*. 1–3. 32647311 10.1038/s41396-020-0712-5 PMC7490696

[B84] Vila-Costa M. Gasol J. M. Sharma S. Moran M. A. (2012). Community analysis of high-and low-nucleic acid-containing bacteria in NW Mediterranean coastal waters using 16S rDNA pyrosequencing. *Environ. Microbiol.* 14 1390–1402. 22390635 10.1111/j.1462-2920.2012.02720.x

[B85] Vila-Costa M. Sebastián M. Pizarro M. Cerro-Gálvez E. Lundin D. Gasol J. M. (2019). Microbial consumption of organophosphate esters in seawater under phosphorus limited conditions. *Sci. Rep.* 9:233. 10.1038/s41598-018-36635-2 PMC6338803 30659251

[B86] Wang Y. Hammes F. Boon N. Chami M. Egli T. (2009). Isolation and characterization of low nucleic acid (LNA)-content bacteria. *ISME J.* 3 889–902. 19421234 10.1038/ismej.2009.46

[B87] Wang Y. Hatt J. K. Tsementzi D. Rodriguez-R L. M. Ruiz-Pérez C. A. Weigand M. R. (2017). Quantifying the importance of the rare biosphere for microbial community response to organic pollutants in a freshwater ecosystem. *Appl. Environ. Microbiol.* 83 3321–3337. 10.1128/AEM.03321-16 PMC5377499 28258138

[B88] Wickham H. (2009). *Ggplot2: Elegant Graphics for Data Analysis*, 2nd Edn New York, NY: Springer.

[B89] Wilkins D. Yau S. Williams T. J. Allen M. A. Brown M. V. Demaere M. Z. (2013). Key microbial drivers in Antarctic aquatic environments. *FEMS Microbiol. Rev.* 37 303–335. 23062173 10.1111/1574-6976.12007

[B90] Wittgens A. Kovacic F. Müller M. M. Gerlitzki M. Santiago-Schübel B. Hofmann D. (2017). Novel insights into biosynthesis and uptake of rhamnolipids and their precursors. *Appl. Microbiol. Biotechnol.* 101 2865–2878. 27988798 10.1007/s00253-016-8041-3 PMC5352749

[B91] Wurl O. Holmes M. (2008). The gelatinous nature of the sea-surface microlayer. *Mar. Chem.* 110 89–97.

[B92] Wurl O. Miller L. Röttgers R. Vagle S. (2009). The distribution and fate of surface-active substances in the sea-surface microlayer and water column. *Mar. Chem.* 115 1–9.

[B93] Wurl O. Obbard J. P. (2004). A review of pollutants in the sea-surface microlayer (SML): a unique habitat for marine organisms. *Mar. Pollut. Bull.* 48 1016–1030. 15172807 10.1016/j.marpolbul.2004.03.016

[B94] Yuan X. Floresyona D. Aubert P. H. Bui T. T. Remita S. Ghosh S. (2019). Photocatalytic degradation of organic pollutant with polypyrrole nanostructures under UV and visible light. *Appl. Catal. B Environ.* 242 284–292.

[B95] Zäncker B. Cunliffe M. Engel A. (2018). Bacterial community composition in the sea surface microlayer off the peruvian coast. *Front. Microbiol.* 9:2699. 10.3389/fmicb.2018.02699 30498480 PMC6249803

[B96] Ẑutic V. Cosovic B. Marcenko E. Bihari N. Kršinic F. (1981). Surfactant production by marine phytoplankton. *Mar. Chem.* 10 505–520.

