# Supplementary material for: Large Enrichment of Anthropogenic Organic Matter Degrading Bacteria in the Sea-Surface Microlayer at Coastal Livingston Island (Antarctica)
Source: Front Microbiol. 2020 Sep 11;11:571983. doi: 10.3389/fmicb.2020.571983 (PMC7516020; doi:10.3389/fmicb.2020.571983)
Supplement: Supplementary file 1 [file Data_Sheet_1.PDF]

Supplementary Material

List of contents

Table S1. Coordinates of the sampling stations from South Bay, Livingston Island.

Table S2. Ancillary data for the seawater sampling sites.

Table S3. Meteorological conditions for each sampling event. The values were averaged coinciding the sampling hours (From 9 am to 11.30 am).

Table S4. Inorganic nutrient concentrations and PFOS concentrations for the SML and SSL and for each sampling event. PFOS is taken as surrogate of ADOC and SML occurrence.

Table S5. Subset of taxa, previously reported as bacterial ADOC degraders (AD), detected in our dataset from the maritime Antarctica. As these taxa have also been reported to exudate biosurfactants, the type of surfactants and citation is also given. In bold, marine biosurfactant-producing bacterial strains.

Figure S1. Nutrient and PFOS concentrations in the SML and SSL during the austral summer. Gray background indicates those days with SML disruption. Average wind speed and solar radiation measurements are also shown.

Figure S2. Relative abundances of the dominant taxonomical groups at the SML and in SSL for each sampling station.

Figure S3. Relative abundance of the dominant taxonomical groups in the SML and SSL during the austral summer. Average wind speed and solar radiation measurements are also shown.

Figure S4. ADOC degraders relative abundance for each layer during the austral summer and corresponding taxonomical class.

Table S1. Coordinates of the sampling stations from South Bay, Livingston Island.

Station	Coordinates	
	S	W
St-1	62º38.792'	60º38.411'
St-2	62º38.346'	60º23.912'
St-3	62º39.425'	60º23.277'
St-4	62º 39.556'	60º 22.132'
St-5	62º37.718'	060º21.40'

Table S2. Ancillary data for the seawater sampling sites.

Date	Sampling station	Fluorescence (RFU)	Salinity (PSU)	Temperature (˚C)	Turbidity (FTU)
08/01/2018	St-4	n.d	n.d	n.d	n.d
16/01/2018	St-4	n.d	n.d	n.d	n.d
18/01/2018	St-3	0.27	33.49	1.56	10.11
19/01/2018	St-4	0.27	33.49	1.56	10.11
20/01/2018	St-2	0.66	34.14	1.60	3.56
22/01/2018	St-4	0.40	33.30	1.42	7.84
24/01/2018	St-2	0.88	33.94	2.23	3.66
25/01/2018	St-4	0.27	33.46	1.71	5.85
27/01/2018	St-2	0.31	33.68	2.43	4.86
29/01/2018	St-4	0.58	33.49	1.87	6.04
03/02/2018	St-2	0.15	34.75	1.59	3.55
05/02/2018	St-4	0.44	33.61	1.47	6.41
08/02/2018	St-1	n.d	n.d	n.d	n.d
12/02/2018	St-2	0.18	33.67	1.76	7.11
16/02/2018	St-4	0.23	33.26	1.78	7.44
20/02/2018	St-2	0.23	33.86	2.93	3.87
23/02/2018	St-4	0.19	33.16	1.82	9.83
28/02/2018	St-5	0.28	33.63	1.14	5.44
01/03/2018	St-1	n.d	n.d	n.d	n.d
Averaged by station					
	St-1	n.d	n.d	n.d	n.d
	St-2	0.4±0.3	34.01±0.41	2.09±0.54	4.43±1.4
	St-3	0.27	33.49	1.56	10.11
	St-4	0.32±0.14	33.38±0.15	1.67±0.17	7.62±1.62
	St-5	0.28	33.63	1.14	5.44

Table S3. Meteorological conditions for each sampling event. The values were averaged coinciding the sampling hours (From 9 am to 11.30 am).

Date	Wind speed (m/s)	Wind direction (°)	Temperature (°C)	Minimum temp. (°C)	Maximum temp. (°C)	Relative humidity (%)	Precipitation (mm)	Atmospheric pressure (hPa)	Radiation (Wm^{-2})
08/01/2018	1.2	250.4	1.9	1.8	2.0	88.6	0.0	1000.9	137.5
11/01/2018	2.0	203.4	2.4	2.2	2.6	95.9	0.0	980.4	86.1
16/01/2018	3.7	56.3	2.7	2.6	2.8	89.9	0.0	978.3	107.4
17/01/2018	0.7	197.3	2.3	2.2	2.5	93.2	0.0	978.8	67.4
18/01/2018	1.3	160.9	1.3	1.1	1.4	88.5	0.0	989.3	98.0
19/01/2018	1.1	156.8	3.0	2.9	3.2	90.9	0.0	978.2	6.7
20/01/2018	n.d.	n.d.	n.d.	n.d.	n.d.	n.d.	n.d.	n.d.	n.d.
22/01/2018	3.1	0.0	1.9	1.8	2.0	92.8	0.0	973.8	29.2
24/01/2018	3.6	18.6	1.8	1.8	1.9	93.9	0.0	985.8	50.7
25/01/2018	1.4	102.5	2.7	2.6	2.8	92.5	0.0	984.5	33.8
27/01/2018	1.5	65.7	2.7	2.6	2.8	75.9	0.0	990.6	153.8
29/01/2018	4.6	53.9	5.6	5.4	5.7	74.3	0.0	979.1	136.6
03/02/2018	1.6	50.8	3.0	3.1	3.0	92.9	0.0	977.2	18.0
05/02/2018	1.0	188.3	2.4	2.3	2.6	88.4	0.0	987	126.8
06/02/2018	6.5	0.0	3.0	2.9	3.5	95.4	0.1	980.9	14.5
08/02/2018	3.9	32.7	2.5	2.4	4.2	93.0	0.0	973.4	23.9
12/02/2018	0.4	131.8	3.4	1.4	3.2	91.1	0.0	974.3	39.1
16/02/2018	4.0	33.5	4.1	3.4	4.0	88.0	0.0	986.4	21.4
20/02/2018	6.7	22	1.4	1.3	1.3	88.4	0.0	997.7	41.8
23/02/2018	1.8	74.4	3.3	3.2	3.2	94.6	0.0	992.0	22.5
28/02/2018	2.8	155.8	0.6	0.6	0.7	84.3	0.0	991.4	38
01/03/2018	1.5	52.1	3.1	3.0	3.3	91.1	0.0	970.1	19.5

Table S4. Inorganic nutrient concentrations and PFOS concentrations for the SML and SSL and for each sampling event. PFOS is taken as surrogate of ADOC and SML occurrence.

Date	Sampling Station	sea layer	**Inorganic nutrients**			**Proxy of ADOC and SML stability**
			N-NH_4^+ (µmolL^{-1})	NO_3^- + NO_2^- (µmolL^{-1})	-PO_4^{3-} (µmol L^{-1})	PFOS (pg L^{-1})
08/01/2018	St-4	sml	n.d	n.d	n.d	n.d
		ssl	7.65±7.42	9.56±1.08	1.06±0.1	n.d
16/01/2018	St-4	sml	2.43	22.21	1.71	85
		ssl	10.8±9.23	19.83±2.87	2.12±0.1	32.5
18/01/2018	St-3	sml	n.d	n.d	n.d	55
		ssl	5.43	14.6	1.26	17.5
19/01/2018	St-4	sml	6.89	14.52	1.68	40
		ssl	n.d	n.d	n.d	17.5
20/01/2018	St-2	sml	1.9	27.04	1.78	15
		ssl	1.97±0.27	26.15±1.39	1.69±0.09	16.25
22/01/2018	St-4	sml	5.24	21.36	1.78	15
		ssl	20.91±20.34	19.37±2.19	1.535±0.19	8.75
24/01/2018	St-2	sml	2.98	26.39	1.52	25
		ssl	2.65±1.14	26.83±0.13	1.87±0.13	8.75
25/01/2018	St-4	sml	n.d	n.d	n.d	60
		ssl	2.84±0.0	18.31±3.41	1.42±0.14	8.75
27/01/2018	St-2	sml	n.d	n.d	n.d	230
		ssl	2.52±1.81	23.27±7.12	1.4±0.06	8.75
29/01/2018	St-4	sml	3.04	24.3	1.87	35
		ssl	3.05±0.49	20.47±3.73	1.59±0.16	10
03/02/2018	St-2	sml	13.39	19.7	1.39	30
		ssl	10.44	16.4	1.68	17.5
05/02/2018	St-4	sml	11.29	18.6	1.89	n.d
		ssl	5.08±1.69	15.32±4.31	1.46±0.11	n.d
08/02/2018	St-1	sml	1.74	27.37	1.93	15
		ssl	3.07±1.48	23.36±3.43	1.62±0.18	12.5
12/02/2018	St-2	sml	1.31	23.08	1.55	20
		ssl	13.05±0.62	20.7±1.5	1.35±0.13	10
16/02/2018	St-4	sml	2.73	19.23	1.52	35
		ssl	9.76±0.58	17.75±1.35	1.32	33.75
20/02/2018	St-2	sml	n.d	n.d	n.d	25
		ssl	6.975±6.47	20.78±2.94	1.38±0.144	7.5
23/02/2018	St-4	sml	7.6±1.84	16.92±0.76	1.40±0.20	n.d
		ssl	8.51±5.34	14.53±3.26	1.26±0.11	n.d
28/02/2018	St-5	sml	n.d	n.d	n.d	n.d
		ssl	5.32±2.34	24.25±0.63	1.39±0.01	n.d
01/03/2018	St-1	sml	n.d	n.d	n.d	70
		ssl	1.12±0.02	10.6±0.75	1.16±0.1	22.5

Averaged by station

St-1	sml	1.72±0.02	25.4±2.28	1.75±0.2	42.5±38.8
	ssl	2.09±1.43	16.98±7.2	1.39±0.28	17.5± 7.1
St-2	sml	4.08±4.96	24.2±2.78	1.65±0.24	64±93
	ssl	5.89±5.07	22.9±4.45	1.55±0.23	12.3±4.2
St-3	sml	2.83	26.79	1.8	55
	ssl	5.43	14.61	1.26	17.5
St-4	sml	5.61±2.99	18.95±3.07	1.64±0.2	43.8±21.9
	ssl	8.57±9.33	16.9±4.32	1.47±0.31	18.5± 11.1
St-5	ssl	5.32±2.34	24.25±0.63	1.39±0.01	n.d
TOTAL AVERAGED	sml	4.3 ± 3.7	22 ± 4.1	1.7 ± 0.2*	50.3±50.8
	ssl	6.3 ± 7	19.1 ± 5.4	1.4 ± 0.3*	16.5±8.7

Table S5. Subset of taxa, previously reported as bacterial ADOC degraders (AD) (Takahashi *et al.*, 2013; Lozada *et al.*, 2014), detected in our dataset from the maritime Antarctica. As these taxa have also been reported to exudate biosurfactants, the type of surfactants and citation is also given. In bold, marine biosurfactant-producing bacterial strains.

Label	Phylum/Class	Genus	Biosurfactant-producing bacteria	
Actinobacteria AD	Phylum: Actinobacteria	*Mycobacterium*	Polymeric surfactants	Vijayakumar and Saravanan, 2015
		Nocardioides		
		Rhodococcus	**Exopolysacharides/complex surface active polymer producer**	Peng *et al.*, 2007
		Arthrobacter	**Glycolipid surface active molecules**	Schulz *et al.*, 1991
		Gordonia	Extracellular bioemulsifiers, and cell-bound glycolipid biosurfactants (isolated from hydrocarbon polluted site)	Franzetti *et al.*, 2009
		Microbacterium	Biosurfactant (long chain fatty acid lipid type) (isolated from Amazonas river)	Silva Lima *et al.*, 2017
		Dietzia	**Rhamnolipid**	Wang *et al.*, 2014
		Micrococcus	Biosurfactant (isolated from waste water sludge)	Yilmaz *et al.*, 2009
Alphaproteobacteria AD	Phylum: Proteobacteria Class: Alphaproteobacteria	*Sulfitobacter*		
		Sphingopyxis	Biosurfactant (Contaminated soil)	Saisa-ard *et al.*, 2013
		Sphingomonas	Polymeric surfactant	Santos *et al.*, 2016
		Roseovarius	**Biosurfactant**	Antoniou *et al.*, 2015
		Roseobacter		
		Sphingobium	Biourfactant (Contaminated soil)	Saisa-ard *et al.*, 2013
		Tropicibacter		
		Novosphingobium		
		Jannaschia		
		Ochrobactrum	Biosurfactant	Zarinviarsagh

				et al., 2017 Domingues *et al.*, 2020
		Thalassospira	**Biosurfactant**	Antoniou *et al.*, 2015
		Kordiimonas		
Bacilli AD	Phylum: Firmicutes Class: Bacilli	*Bacillus*	**Exopolysacharide, complex surface active polymer/polymeric surfactants**	Das *et al.*, 2008
Betaproteobacteria AD	Phylum: Proteobacteria Class: Betaproteobacteria	*Acidovorax*		
		Comamonas	Biosurfactant (From agricultural soil)	Sun *et al.*, 2013
		Polaromonas		
		Ralstonia	Biosurfactant (from Petroleum waste)	Płaza *et al.*, 2007
		Delftia	glycolipids Surfactant (from Oilfield water injection)	Lenchi *et al.*, 2020
		Alcaligenes	Biosurfactant (from Petroleum waste)	Płaza *et al.*, 2007
Cytophagia AD	Phylum: Bacteroidetes Class: Cytophagia	*Cytophaga*		
Deltaproteobacteria AD	Phylum: Proteobacteria Class: Deltaproteobacteria	*Desulfatiferula*		
		Desulfococcus		
		Desulfatibacillum		
		Desulfobacterium		
Flavobacteriia AD	Phylum: Bacteroidetes Class: Flavobacteriia	*Flavobacterium*	Biosurfactant (From contaminated arid soils)	Bodour *et al.*, 2003
Gammaproteobacteria AD	Phylum: Proteobacteria Class: Gammaproteobacteria	*Pseudoalteromonas*	**Exopolysacharides/complex Surface active polymer**	Roca *et al.*, 2016
		Pseudomonas	**Polymeric biosurfactant/bioemulsifier, lipopeptides, glycolipids and particulate surfactant**	Wittgens *et al.*, 2017
		Colwellia	**Biosurfactant**	Mapelli *et al.*, 2017
		Cycloclasticus	**Biosurfactant**	Mapelli *et al.*, 2017
		Alkanindiges		
		Acinetobacter	**Polymeric biosurfactant/bioemulsifier**	Rosenberg *et al.*, 1988
		Oleispira		
		Shewanella	**Biosurfactant**	Antoniou *et al.*, 2015
		Halomonas	**Polymeric biosurfactant/bioemulsifier,**	Gutiérrez *et al.*, 2007

		**glycolipid surface active molecules and Exopolysacharides (complex Surface active polymer)**	
	Vibrio	**Exopolysacharides (complex Surface active polymer)**	Satpute *et al.*, 2010
	Oleibacter		
	Alteromonas	Biosurfactant	Satpute *et al.*, 2010
	Microbulbifer		
	Neptunomonas		
	Thalassolituus		
	Marinobacter	**Polymeric biosurfactant/bioemulsifier**	Domingues et al 2020
	Alcanivorax	**Glycolipid surface active molecules (rhamnolipid and sophorolipid)**	Abraham *et al.*, 1998
All AD	*Includes all listed above		

Figure S1. Nutrient and PFOS concentrations in the SML and SSL during the austral summer. Gray background indicates those days with SML disruption. Average wind speed and solar radiation measurements are also shown.

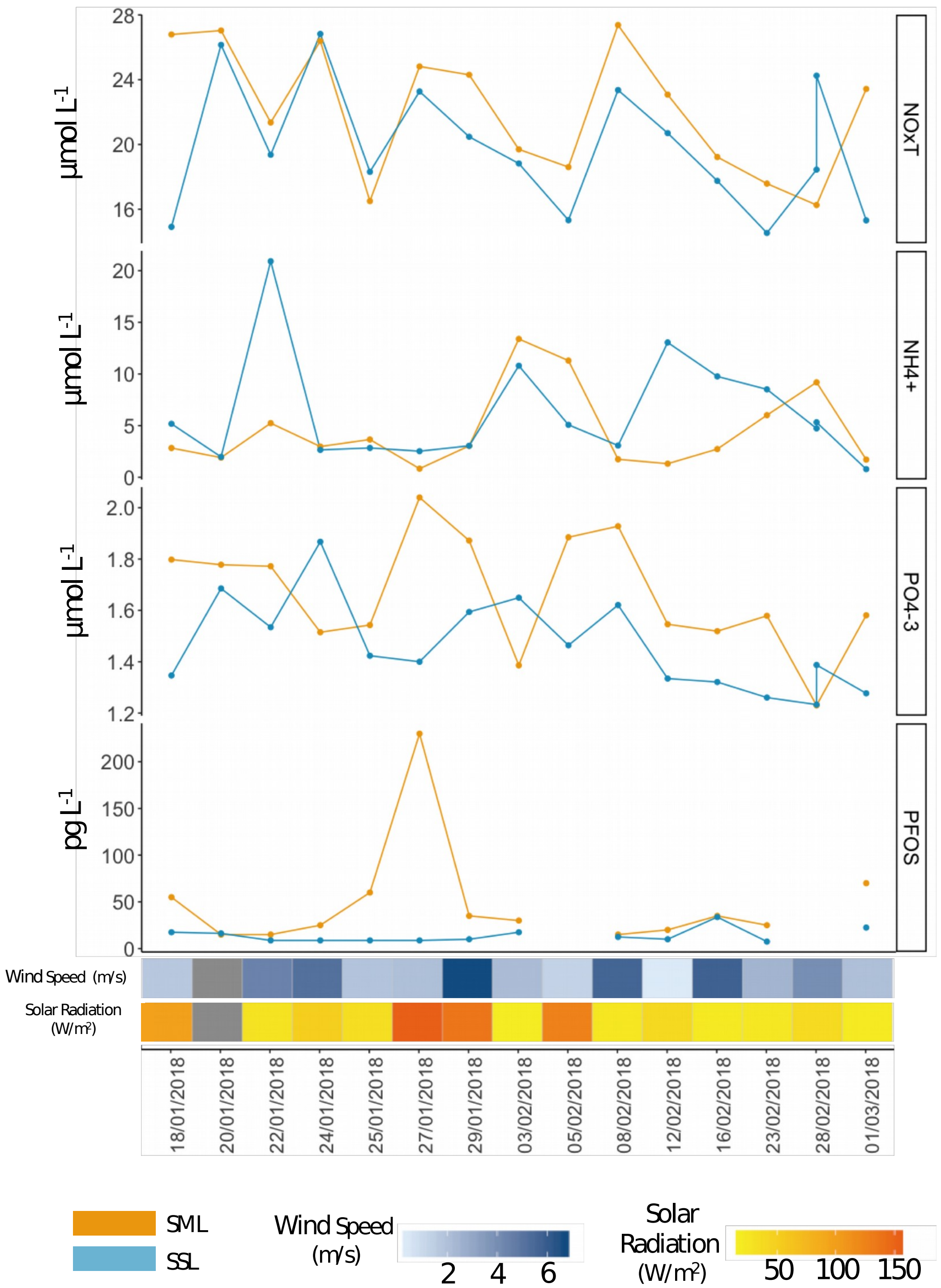

Figure S2. Relative abundances of the dominant taxonomical groups at the SML and in SSL for each sampling station.

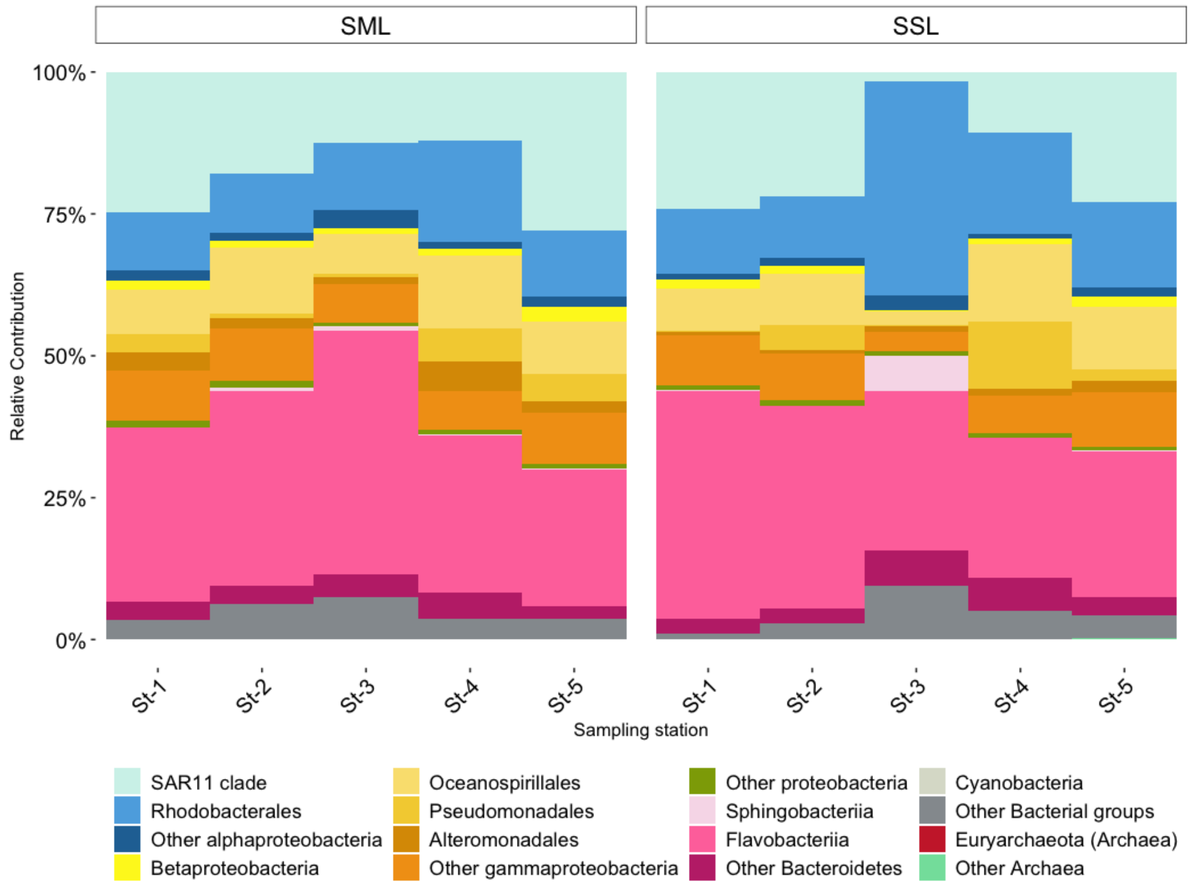

Figure S3. Relative abundance of the dominant taxonomical groups in the SML and SSL during the austral summer. Average wind speed and solar radiation measurements are also shown.

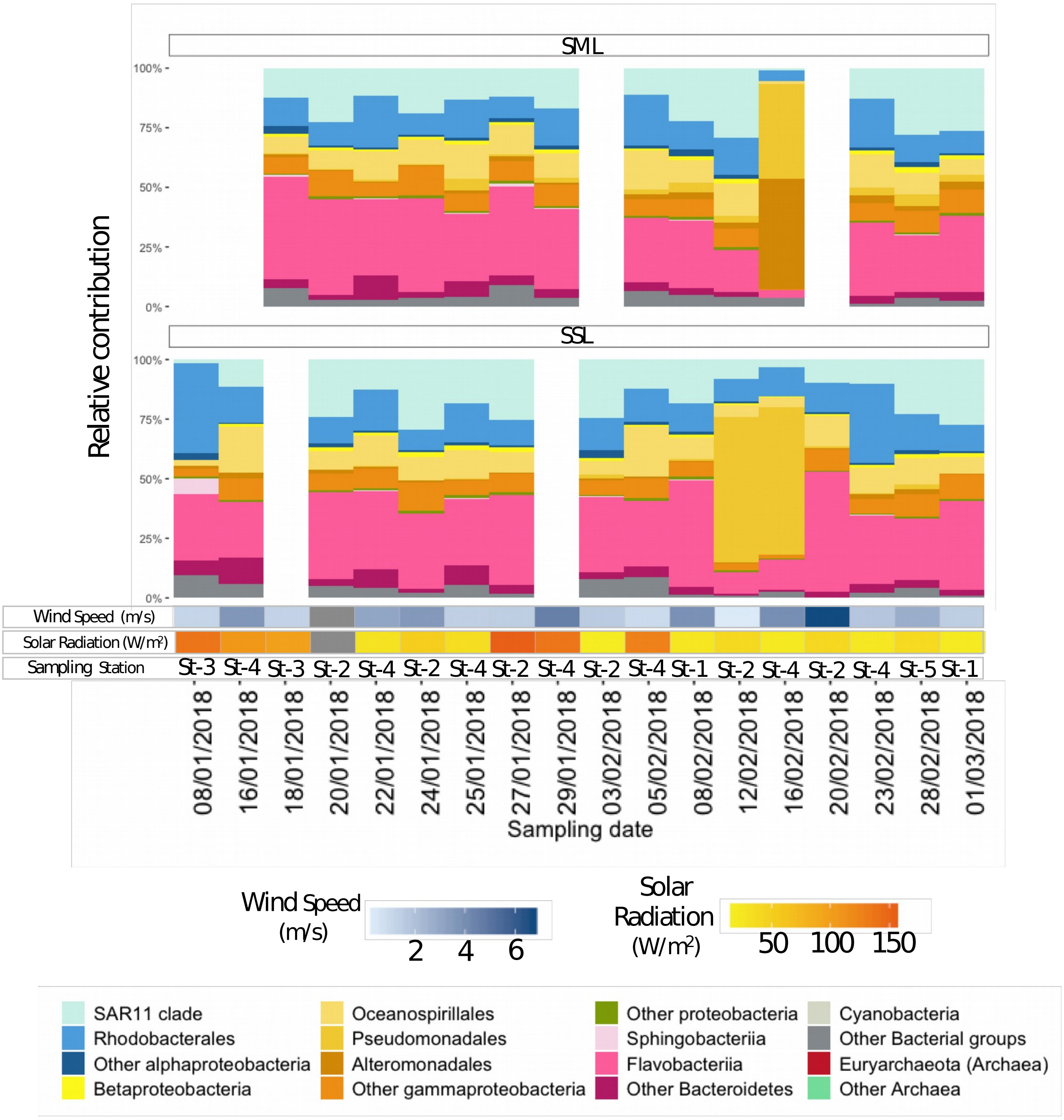

Figure S4. ADOC degraders relative abundance for each layer during the austral summer and corresponding taxonomical class.

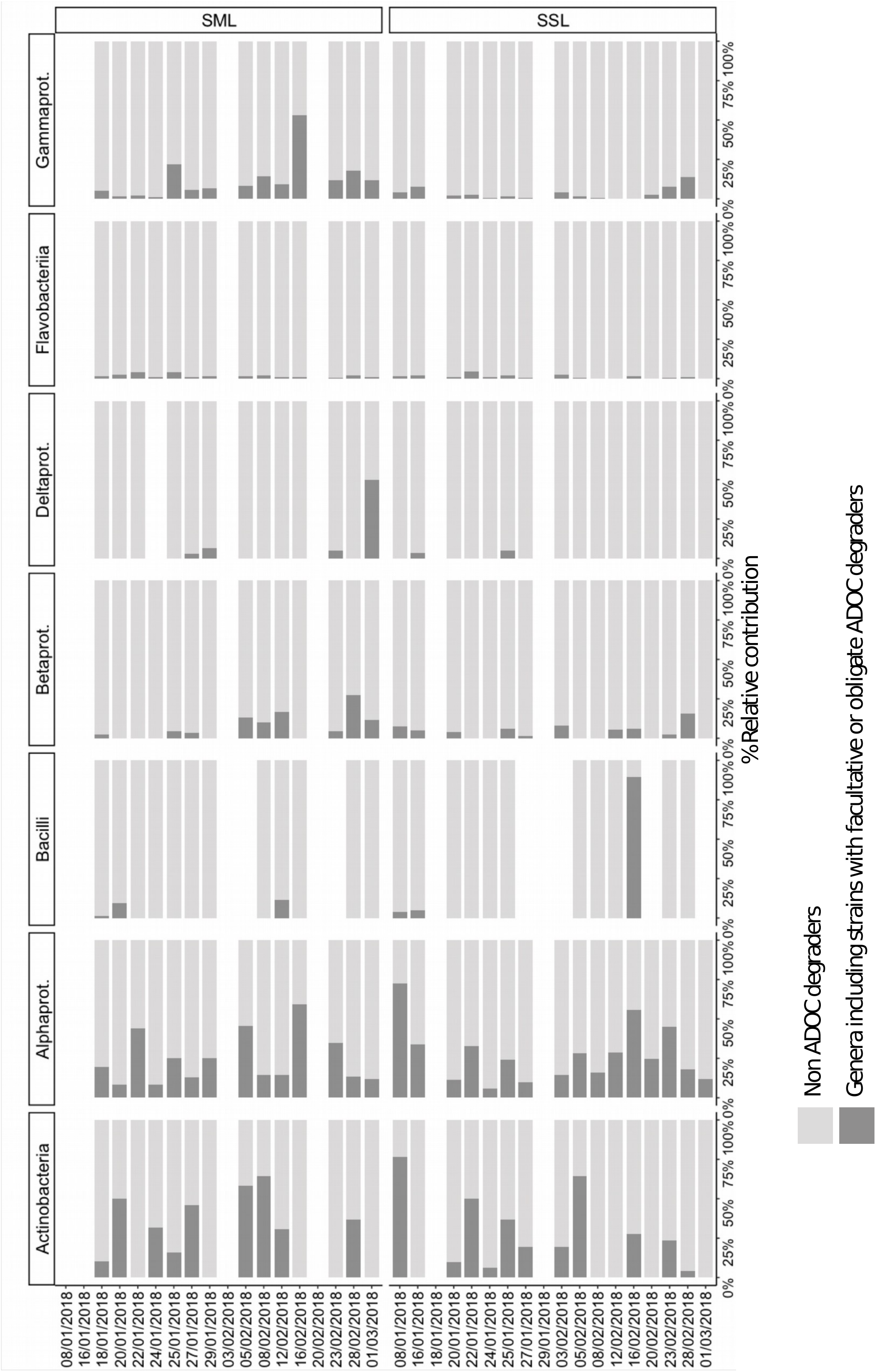

References

Abraham, W.R., Meyer, H., and Yakimov, M. (1998) Novel glycine containing glucolipids from the alkane using bacterium Alcanivorax borkumensis. Biochim Biophys Acta - Lipids Lipid Metab 1393: 57–62.

Antoniou, E., Fodelianakis, S., Korkakaki, E., and Kalogerakis, N. (2015) Biosurfactant production from marine hydrocarbon-degrading consortia and pure bacterial strains using crude oil as carbon source. Front Microbiol 6: 274.

Bodour, A.A., Drees, K.P., and Maier, R.M. (2003) Distribution of biosurfactant-producing bacteria in undisturbed and contaminated arid southwestern soils. Appl Environ Microbiol 69: 3280–3287.

Das, P., Mukherjee, S., and Sen, R. (2008) Improved bioavailability and biodegradation of a model polyaromatic hydrocarbon by a biosurfactant producing bacterium of marine origin. Chemosphere 72: 1229–1234.

Domingues, P.M., Oliveira, V., Serafim, L.S., Gomes, N.C.M., and Cunha, Â. (2020) Biosurfactant production in sub-oxic conditions detected in hydrocarbon-degrading isolates from marine and estuarine sediments. Int J Environ Res Public Health 17:.

Franzetti, A., Caredda, P., La Colla, P., Pintus, M., Tamburini, E., Papacchini, M., and Bestetti, G. (2009) Cultural factors affecting biosurfactant production by Gordonia sp. BS29. Int Biodeterior Biodegrad 63: 943–947.

Gutiérrez, T., Mulloy, B., Black, K., and Green, D.H. (2007) Glycoprotein emulsifiers from two marine Halomonas species: Chemical and physical characterization. J Appl Microbiol 103: 1716–1727.

Lenchi, N., Kebbouche-Gana, S., Servais, P., Gana, M.L., and Llirós, M. (2020) Diesel Biodegradation Capacities and Biosurfactant Production in Saline-Alkaline Conditions by Delftia sp NL1, Isolated from an Algerian Oilfield. Geomicrobiol J. 37: 454-466.

Lozada, M., Marcos, M.S., Commendatore, M.G., Gil, M.N., and Dionisi, H.M. (2014) The bacterial community structure of hydrocarbon-polluted marine environments as the basis for the definition of an ecological index of hydrocarbon exposure. Microbes Environ 29: 269–76.

Peng, F., Liu, Z., Wang, L., and Shao, Z. (2007) An oil-degrading bacterium: Rhodococcus erythropolis strain 3C-9 and its biosurfactants. J Appl Microbiol 102: 1603–1611.

Płaza, G.A., Jangid, K., Łukasik, K., Nałęcz-Jawecki, G., Berry, C., and Brigmon, R.L. (2007) Biodegradation of petroleum-waste by biosurfactant-producing bacteria, Savannah River Site (S.C.).

Roca, C., Lehmann, M., Torres, C.A.V., Baptista, S., Gaudêncio, S.P., Freitas, F., and Reis, M.A.M. (2016) Exopolysaccharide production by a marine Pseudoalteromonas sp. strain isolated from Madeira Archipelago ocean sediments. N Biotechnol 33: 460–466.

Rosenberg, E., Rubinovitz, C., Legmann, R., and Ron, E. (1988) urification and chemical properties of Acinetobacter calcoaceticus A2 biodispersan. Appl Env Microbiol. 54 : 323-326.

Saisa-Ard, K., Saimmai, A., and Maneerat, S. (2013) Characterization and phylogenetic analysis of biosurfactant-producing bacteria isolated from palm oil contaminated soils. Songklanakarin Journal of Science & Technology, 36.

Santos, D.K.F., Rufino, R.D., Luna, J.M., Santos, V.A., and Sarubbo, L.A. (2016) Biosurfactants: multifunctional biomolecules of the 21st century. Int J Mol Sci 17: 401.

Satpute, S.K., Banat, I.M., Dhakephalkar, P.K., Banpurkar, A.G., and Chopade, B.A. (2010) Biosurfactants, bioemulsifiers and exopolysaccharides from marine microorganisms. Biotechnol Adv 28: 436–450.

Schulz, D., Passeri, A., Schmidt, M., Lang, S., Wagner, F., Wray, V., and Gunkel, W. (1991) Marine biosurfactants, I. Screening for biosurfactants among crude oil degrading marine microorganisms from the North Sea. Z Naturforsch 46: 197-203.

Silva Lima, J.M., Pereira, J.O., Batista, I.H., Pereira Junior, R.C., Barroso, H. dos S., Neto, P. de Q.C., et al. (2017) Biossurfactantes produzidos por Microbacterium sp., isolada de macrófita aquática em área impactada por hidrocarbonetos no Rio Negro, Manaus, Amazonas. Acta Sci - Biol Sci 39: 13–20.

Sun, L.N., Zhang, J., Chen, Q., He, J., Li, Q.F., and Li, S.P. (2013) Comamonas jiangduensis sp. nov., a biosurfactantproducing bacterium isolated from agricultural soil. Int J Syst Evol Microbiol 63: 2168–2173.

Takahashi, S., Abe, K., and Ker, Y. (2013) Microbial Degradation of Persistent Organophosphorus Flame Retardants. In Environmental Biotechnology - New Approaches and Prospective Applications. InTech. Edited by Marian Petre: 91.

Vijayakumar, S. and Saravanan, V. (2015) Biosurfactants-Types, Sources and Applications. Res J Microbiol 10: 181–192.

Wang, W., Cai, B., and Shao, Z. (2014) Oil degradation and biosurfactant production by the deep sea bacterium Dietzia maris As-13-3. Front Microbiol 5: 711.

Wittgens, A., Kovacic, F., Müller, M.M., Gerlitzki, M., Santiago-Schübel, B., Hofmann, D., et al. (2017) Novel insights into biosynthesis and uptake of rhamnolipids and their precursors. Appl Microbiol Biotechnol 101: 2865–2878.

Yilmaz, F., Ergene, A., Yalcin, E., and Tan, S. (2009) Production and characterization of biosurfactants produced by microorganisms isolated from milk factory wastewaters. Environ Technol 30: 1397–1404.

Zarinviarsagh, M., Ebrahimipour, G., and Sadeghi, H. (2017) Lipase and biosurfactant from Ochrobactrum intermedium strain MZV101 isolated by washing powder for detergent application. Lipids Health Dis 16: 177.
